# The Maternal Maverick/GDF15-like TGF-β Ligand Panda Directs Dorsal-Ventral Axis Formation by Restricting Nodal Expression in the Sea Urchin Embryo

**DOI:** 10.1371/journal.pbio.1002247

**Published:** 2015-09-09

**Authors:** Emmanuel Haillot, Maria Dolores Molina, François Lapraz, Thierry Lepage

**Affiliations:** Institut de Biologie Valrose, iBV, UMR 7277 CNRS, Inserm U1091, UNS, University of Nice Sophia Antipolis, Nice, France; The Francis Crick Institute, UNITED KINGDOM

## Abstract

Specification of the dorsal-ventral axis in the highly regulative sea urchin embryo critically relies on the zygotic expression of *nodal*, but whether maternal factors provide the initial spatial cue to orient this axis is not known. Although redox gradients have been proposed to entrain the dorsal-ventral axis by acting upstream of *nodal*, manipulating the activity of redox gradients only has modest consequences, suggesting that other factors are responsible for orienting *nodal* expression and defining the dorsal-ventral axis. Here we uncover the function of Panda, a maternally provided transforming growth factor beta (TGF-β) ligand that requires the activin receptor-like kinases (Alk) Alk3/6 and Alk1/2 receptors to break the radial symmetry of the embryo and orient the dorsal-ventral axis by restricting *nodal* expression. We found that the double inhibition of the bone morphogenetic protein (BMP) type I receptors Alk3/6 and Alk1/2 causes a phenotype dramatically more severe than the BMP2/4 loss-of-function phenotype, leading to extreme ventralization of the embryo through massive ectopic expression of *nodal*, suggesting that an unidentified signal acting through BMP type I receptors cooperates with BMP2/4 to restrict *nodal* expression. We identified this ligand as the product of maternal Panda mRNA. Double inactivation of *panda* and *bmp2/4* led to extreme ventralization, mimicking the phenotype caused by inactivation of the two BMP receptors. Inhibition of maternal *panda* mRNA translation disrupted the early spatial restriction of *nodal*, leading to persistent massive ectopic expression of *nodal* on the dorsal side despite the presence of Lefty. Phylogenetic analysis indicates that Panda is not a prototypical BMP ligand but a member of a subfamily of TGF-β distantly related to Inhibins, Lefty, and TGF-β that includes Maverick from *Drosophila* and GDF15 from vertebrates. Indeed, overexpression of Panda does not appear to directly or strongly activate phosphoSmad1/5/8 signaling, suggesting that although this TGF-β may require Alk1/2 and/or Alk3/6 to antagonize *nodal* expression, it may do so by sequestering a factor essential for Nodal signaling, by activating a non-Smad pathway downstream of the type I receptors, or by activating extremely low levels of pSmad1/5/8. We provide evidence that, although *panda* mRNA is broadly distributed in the early embryo, local expression of *panda* mRNA efficiently orients the dorsal-ventral axis and that Panda activity is required locally in the early embryo to specify this axis. Taken together, these findings demonstrate that maternal *panda* mRNA is both necessary and sufficient to orient the dorsal-ventral axis. These results therefore provide evidence that in the highly regulative sea urchin embryo, the activity of spatially restricted maternal factors regulates patterning along the dorsal-ventral axis.

## Abstract 2

Panda, a member of the TGF-beta family of signaling molecules, is encoded by maternal mRNA and helps to break radial symmetry and orient the dorsal-ventral axis of the developing sea urchin embryo.

## Author Summary

A key event during development of bilaterians is specification of the anterior-posterior and dorsal-ventral axes of the embryo. In some species, such as the fly *Drosophila*, this process relies on the activity of maternal determinants localized into the egg during oogenesis. However, in other animals, such as mammals or echinoderms, which are renowned for the developmental plasticity of their embryos, there is presently no evidence for maternal determinants controlling axis formation, and how these embryonic axes emerge from radially symmetrical embryos remains unknown. In the sea urchin embryo, specification of the dorsal-ventral axis critically relies on the localized expression of the TGF-β ligand Nodal in the presumptive ventral territory, but what controls the spatially restricted expression of *nodal* is not known. We discovered that in the sea urchin embryo, the initial restriction of *nodal* expression is directed by another TGF-β ligand that is expressed maternally, which we named Panda. Panda is both necessary for the early spatial restriction of *nodal* and sufficient to orient the dorsal-ventral axis when misexpressed locally. Altogether, our findings suggest that Panda may act as a maternal signal that defines the orientation of the dorsal-ventral axis. Thus, an antagonism between Nodal and maternal Panda signaling drives dorsal-ventral axis formation in the sea urchin embryo.

## Introduction

In bilaterians, specification of the dorsal-ventral (D/V) axis is a crucial event during embryogenesis to establish the correct body plan. In many species, this process relies on gene products translated from maternal mRNAs deposited in the egg. For example, in *Drosophila*, specification of the D/V axis of the embryo is initiated by the product of the *gurken* gene, which is active in the oocyte nucleus during oogenesis and encodes a member of the epidermal growth factor (EGF) superfamily that acts as a secreted dorsalizing signal [1–4]. Similarly, in *Xenopus* and zebrafish, although the D/V axis is not preformed in the unfertilized egg, dorsal determinants are localized to the vegetal pole of the egg [5–8]. Fertilization breaks the radial symmetry of the egg and triggers the asymmetric transport of these determinants from the vegetal pole to the future dorsal side where they activate the canonical Wnt pathway [9,10]. While maternal information is clearly important for specification of the D/V axis in a number of species, in contrast, there is very little evidence for the presence of maternal determinants of axis formation in the oocyte of mammals, consistent with the idea that the embryonic axes are specified entirely by cell interactions [11]. Accordingly, it has been argued that the regulative abilities of the first blastomeres of the mouse embryo rule out the possibility that maternal determinants influence axis specification [12] (reviewed in [13]).

The sea urchin embryo is well known for its extraordinary developmental plasticity [14]. In a now classical blastomere dissociation experiment, Driesch showed that dissociated blastomeres of the four-cell stage embryo have the potentiality to reestablish a D/V axis [15]. The outcome of this experiment not only demonstrated the impressive regulative ability of the early blastomeres of the sea urchin embryo but also strongly influenced ideas about how D/V patterning is established in this organism. By showing that the D/V axis is very easily respecified, it encouraged the view that there are no determinants for D/V axis formation in echinoderm embryos. On the other hand, egg bisection experiments performed by Hörstadius showed that differences in the fates of presumptive ventral and dorsal regions can be traced back to the egg, consistent with the idea that the oocyte already has a bilateral organization [16]. If there are maternal cues that influence D/V axis formation in this embryo, what could they be? There is a large body of evidence correlating formation of the D/V axis with the activity of redox gradients and with the asymmetric distribution of mitochondria in the unfertilized sea urchin egg. Classical experiments performed by Child, Pease, and Czihak in the thirties and sixties showed that it is possible to bias the D/V axis by treating embryos with steep gradients of respiratory inhibitors and that the activity of the mitochondrial enzyme cytochrome oxidase can predict the D/V axis as early as the eight-cell stage, with the presumptive ventral side being more oxidizing than the dorsal side [17–20]. This asymmetry of mitochondria activity is the first known manifestation of D/V polarity. Several recent studies by Coffman and colleagues addressed the question of causality between this early asymmetry and the orientation of the D/V axis [21–23]. Although these studies provided evidence that the D/V axis can be entrained by centrifugation, by microinjection of purified mitochondria, or by overexpressing a form of catalase targeted to the mitochondria, the correlations obtained remained modest, and in no case were these perturbations shown to efficiently orient the D/V axis [21–23]. Furthermore, perturbations that were expected to influence D/V axis formation, such as overexpression of a mitochondrially targeted form of superoxide dismutase, which generates the strong oxidizing component H_2_O_2_ and that would be predicted to efficiently orient the axis, did not show any effect on the orientation of the D/V axis. Therefore, the redox gradient model of D/V axis formation clearly needs further experimental validation, and the biological significance of the early asymmetry of mitochondria and of redox gradients and their relation to the D/V axis remains largely unclear.

At the molecular level, the earliest sign of specification of the D/V axis is the expression of the TGF-β *nodal* in the presumptive ectoderm at the 32-cell stage. *nodal* is the first known zygotic gene differentially expressed along the D/V axis, and Nodal signaling orchestrates patterning along the secondary axis first by specifying the ventral ectoderm and second by inducing the expression of BMP2/4, which acts as a relay to specify the dorsal ectoderm. *nodal* morphants completely lack D/V polarity, but expression of *nodal* into one blastomere is capable of completely rescuing D/V polarity in these embryos [24,25]. *cis*-regulatory studies further showed that *nodal* expression is driven by ubiquitously expressed maternal factors such as the transcription factor SoxB1 and that it requires maternal Wnt/beta catenin signaling as well as signaling by the Vg1/GDF1-related maternal factor Univin [26,27]. Intriguingly, *nodal* is initially expressed very broadly, almost ubiquitously, and then its expression is progressively restricted to a more discrete region of the ectoderm during cleavage. The progressive spatial restriction of *nodal* expression is thought to rely mostly, if not exclusively, on the ability of Nodal to promote its own expression through an intronic autoregulatory enhancer [26,27] and to induce the expression of the long-range Nodal antagonist Lefty. This regulatory mechanism based on the long-range diffusion of the Lefty antagonist fulfills the requirement for a reaction diffusion and is thought to be mainly responsible for amplifying an initial subtle asymmetry, possibly generated by redox gradients, into a robust spatially restricted expression of *nodal* [23,28].

Finally, *nodal* expression requires the integrity of the p38 pathway [22,23,26,29]. Inhibition of p38 signaling with pharmacological inhibitors abolishes *nodal* expression. Immunostaining experiments using an anti-phosho p38 and analysis of the spatial distribution of a p38-green fluorescent protein (GFP) fusion protein revealed that p38 is first activated ubiquitously and then selectively inactivated on the presumptive dorsal side of the embryo. The signals that regulate p38 in the early embryo are not known, but it has been proposed that redox gradients may be directly responsible for p38 activation [22,23,26,29]. However, direct evidence that p38 mediates the effects of redox gradients is presently lacking, and the transcription factors linking p38 to the machinery that regulates *nodal* expression are not known.

In this paper, we identify a maternal factor that plays a crucial role in D/V axis formation by directing the spatial restriction of *nodal* expression. First, we discovered that an unidentified TGF-β ligand cooperates with BMP2/4 to restrict *nodal* expression. We identified this ligand as the product of a maternally expressed TGF-β ligand related to Maverick from Drosophila and GDF15 from vertebrates that we named Panda. Inhibition of maternal *panda* mRNA translation blocked the early spatial restriction of *nodal* and caused persistent massive ectopic expression of *nodal* on the dorsal side despite the presence of Lefty. We further show that while blocking translation of *bmp2/4* mRNA alone does not cause ectopic expression of *nodal*, the double knockdown of *panda* and *bmp2/4* causes an extreme ventralization. We further provide evidence that the *panda* mRNA is broadly distributed in the early embryo, that local expression of *panda* mRNA efficiently orients the D/V axis, and that, although *panda* mRNA is broadly distributed, Panda activity is required locally in the early embryo. Taken together, these findings demonstrate that maternal *panda* mRNA is required early to restrict the spatial expression of *nodal*, that it is sufficient to orient the D/V axis when misexpressed, and therefore, that it fulfills the requirements for a maternal factor that specifies the D/V axis.

Our results suggest that, although specification of the D/V axis is established by the activity of Nodal in the zygote, maternally provided signaling molecules play crucial roles by antagonizing the activity of Nodal.

## Results

### The Type-I BMP Receptor Alk1/2 Is a Central Player in D/V Axis Formation and BMP2/4 Signaling

**Fig 1:**
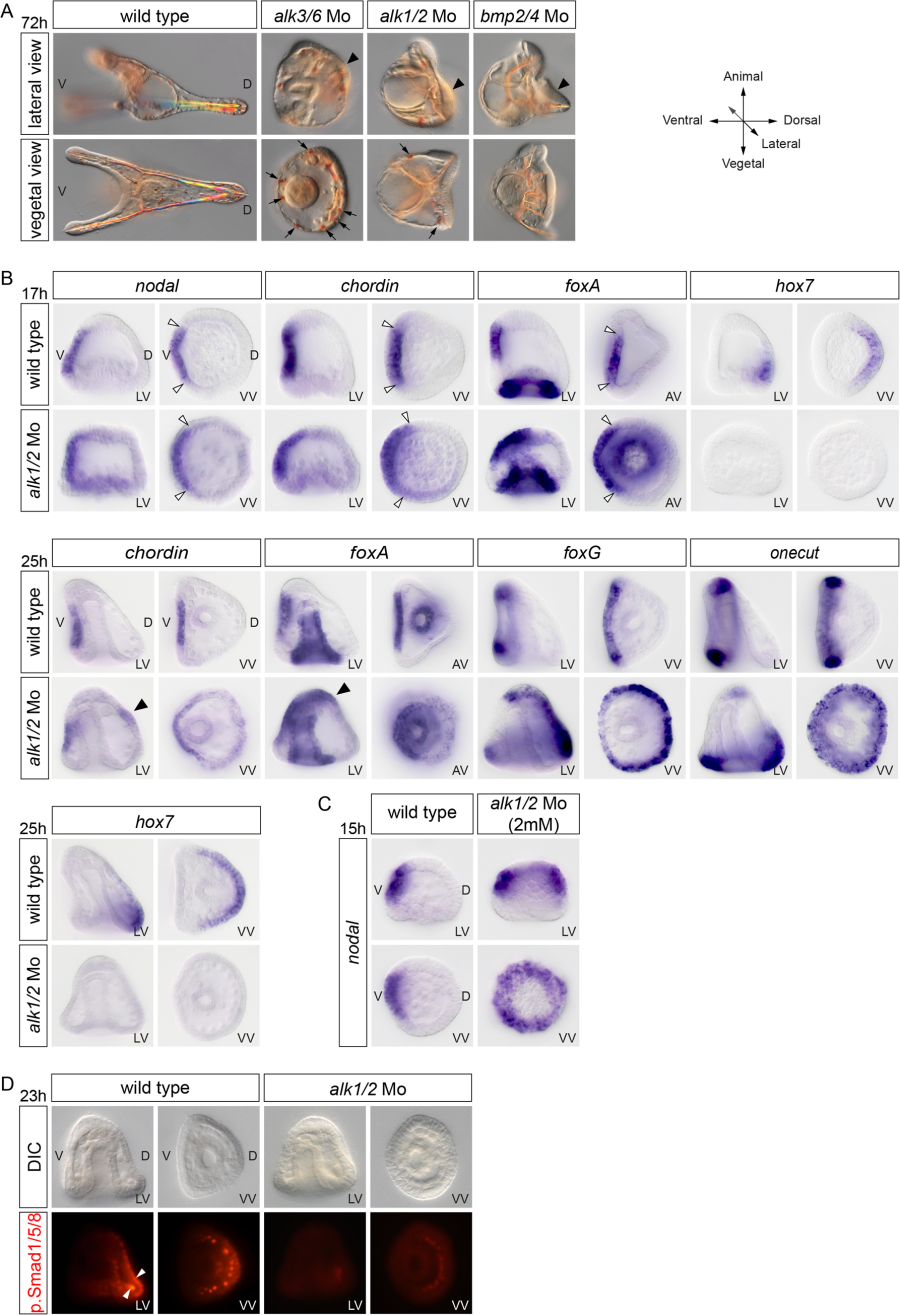
The BMP type I receptor Alk1/2 is essential for D/V patterning. (A) Morphology of embryos at 72 hours after fertilization (hpf) injected with morpholinos targeting either the *alk3/6*, *alk1/2*, or *bmp2/4* transcripts. Note the striking similarity of the phenotypes of *alk1/2* and *bmp2/4* morphants that both develop with a ciliary band on the dorsal side (black arrowheads) compared to the less severe phenotype of *alk3/6* morphants that is evidenced by the presence of pigment cells (black arrows) and of a less well-developed ciliary band on the dorsal side. (B) Expansion of the ventral and ciliary band fates at the expense of the dorsal ectoderm in *alk1/2* morphants was revealed by the analysis of marker genes. Controls and *alk1/2* morphants embryos were stained by in situ hybridization with the indicated probes. In *alk1/2* morphants at mesenchyme blastula, the territory expressing the ventral marker genes, *nodal*, *chordin*, and *foxA* is largely normal, but consistently, a slight broadening of *nodal* expression is observed (white arrowheads), while expression of the dorsal gene *hox7* is suppressed. At the gastrula stage, however, this ventralization is patent with *chordin* and *foxA* expression extending towards the dorsal side in *alk1/2* morphants (black arrowheads). Also note the dramatic dorsal expansion of the ciliary band genes *foxG* and *onecut* in the *alk1/2* morphants. (C) Injection of high doses (2 mM) of *alk1/2* morpholino caused a massive ectopic expression of *nodal* in about 50% of the embryos at the mesenchyme blastula stage. (D) Phospho-Smad1/5/8 immunostaining in control or *alk1/2* morphants. p.Smad1/5/8 in the ectoderm and in the dorsal chain of primary mesenchyme cells (PMCs) (white arrowheads) of *alk1/2* morphants is largely abolished. LV, lateral view; VV, vegetal pole view; AV, animal pole view; D, dorsal; V, ventral.

We showed previously that during D/V patterning in the sea urchin embryo, transduction of the BMP2/4 signals requires the activity of the type-I BMP receptor Alk3/6, the functional orthologue of Thickveins, which transduces Dpp signals in *Drosophila*. We noticed, however, that blocking Alk3/6 consistently produced a phenotype much less severe than the BMP2/4 loss-of-function phenotype. For example, while *bmp2/4* morphants typically lack a population of immunocytes called pigment cells that requires BMP signaling, *alk3/6* morphants always develop with numerous pigments cells (arrows in Fig 1A). This suggested that residual BMP signaling in *alk3/6* morphants allows formation of pigment cells and/or that additional BMP type I receptors may contribute to transduction of BMP2/4 signals in the absence of Alk3/6. Indeed, in addition to *alk3/6*, the sea urchin genome contains a second gene encoding a BMP type I receptor named Alk1/2, which is mostly similar to Alk1 and Alk2 from vertebrates and to Saxophone from *Drosophila*. Like *alk3/6*, *alk1/2* is expressed maternally and ubiquitously during the cleavage and blastula stages (S1 Fig). To evaluate the contribution of Alk1/2 in BMP2/4 signaling, we knocked it down with antisense morpholinos. Interestingly, blocking *alk1/2* mRNA translation disrupted D/V axis formation and produced a phenotype stronger than that resulting from inhibition of Alk3/6 (Fig 1A). When the *alk1/2* morpholino was injected at 1.2 mM, most *alk1/2* morphants failed to develop their ventral arms and dorsal apex and appeared rounded. Alk1/2 morphants also lacked most pigment cells and developed with an ectopic ciliary band and ectopic spicules on the dorsal side, a phenotype largely identical to the *bmp2/4* morphant phenotype. These phenotypes could be suppressed by coinjection of a modified wild-type *alk1/2* mRNA immune against the morpholino (see S1 Fig). As shown previously in the case of Alk3/6 and of BMP2/4, blocking Alk1/2 caused a dramatic expansion of the ciliary band territory at the expense of the dorsal ectoderm, as evidenced by the massive ectopic expression of *foxG* and *onecut* on the presumptive dorsal side and the lack of expression of dorsal marker genes such as *hox7* (Fig 1B). Unexpectedly, blocking Alk1/2 function, unlike blocking BMP2/4 or Alk3/6, caused a weak but consistent ventralization, as evidenced by the expression of *chordin* or *foxA* that extended to the dorsal side at the gastrula stage (black arrowheads in Fig 1B). Consistent with this ventralization, we found that at blastula stages, embryos injected with high doses of the *alk1/2* morpholino displayed a massive ectopic expression of *nodal* similar to that observed in *lefty* morphants (Fig 1C). This phenotype, which is not observed in *bmp2/4* or *alk3/6* morphants, suggests that, in addition to BMP2/4, Alk1/2 may also be required to transduce an unidentified dorsalizing signal. Finally, consistent with the absence of expression of dorsal marker genes, inhibition of *alk1/2* mRNA translation, like inhibition of *bmp2/4* or *alk3/6*, drastically reduced phospho-Smad1/5/8 signaling in the dorsal ectoderm (Fig 1D). We conclude that Alk1/2 plays a pivotal role in transduction of BMP2/4 in the sea urchin and that the activities of Alk1/2 and Alk3/6 are nonredundant, both being functionally required during D/V patterning to transduce BMP2/4 signals and to activate Smad1/5/8 signaling in the dorsal ectoderm. Furthermore, these results suggest that in addition to BMP2/4, Alk1/2 may be required for transduction of (a) still unidentified signal(s) that regulate(s) D/V patterning.

### An Unidentified TGF-β Ligand Acting through Alk1/2 and Alk3/6 Cooperates with BMP2/4 to Restrict *nodal* Expression

**Fig 2:**
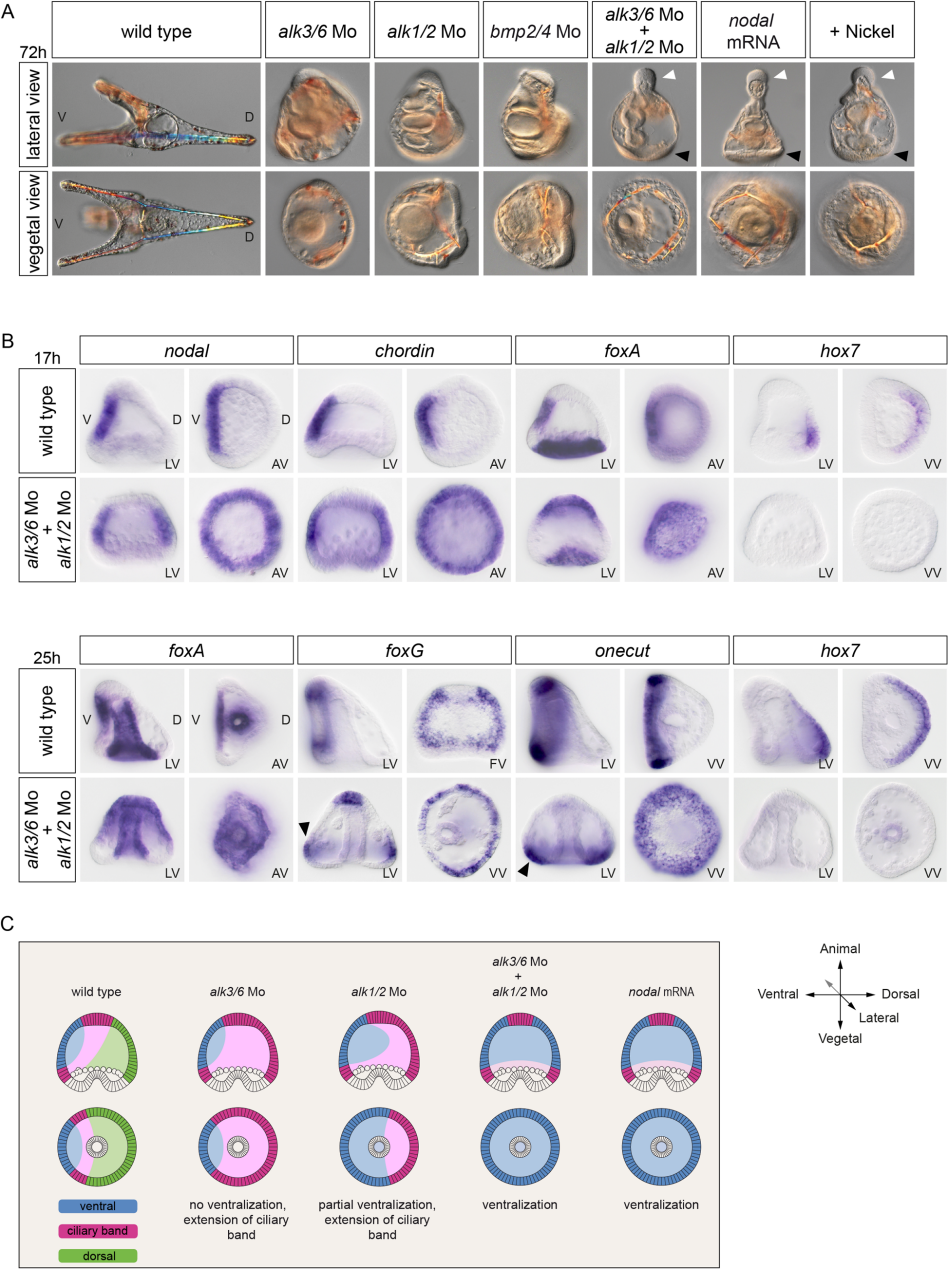
The double inactivation of *alk1/2* and *alk3/6* causes massive ectopic expression of *nodal*, resulting in extreme ventralization. (A) Morphology of embryos at 72 hpf injected with morpholinos targeting either the *alk3/6*, *alk1/2*, or *bmp2/4* transcripts or injected with a mixture of the *alk1/2* and *alk3/6* morpholinos. Simultaneous down-regulation of Alk1/2 and Alk3/6 caused a strong ventralization similar to that resulting from overexpression of *nodal* or from treatment with nickel chloride (a treatment that ventralizes sea urchin embryos by causing massive ectopic expression of *nodal*). Note the presence of a ciliary band in the vegetal pole region (black arrowheads) and the prominent proboscis (white arrowheads) in the animal pole region in the double *alk1/2* + *alk3/6* morphants and in *nodal*-overexpressing or nickel-treated embryos. (B) In situ hybridization on controls and *alk1/2* + *alk3/6* morphants at the blastula and gastrula stages with ventral, ciliary band, and dorsal marker genes. The strong ventralization of *alk1/2* + *alk3/6* morphants is presaged by the massive ectopic expression of *nodal* at blastula stages. Note the radial expression of the ciliary band markers *foxG* and *onecut* in the vegetal pole region of *alk1/2* + *alk3/6* morphants at the gastrula stage. (C) Scheme describing the changes in fate maps caused by the single or double inactivation of type I BMP receptors. In the simple *alk3/6* knockdown, the ventral ectoderm remains unaffected and the dorsal ectoderm is converted into ciliary band, while in the *alk1/2* morphants, the ventral ectoderm is expanded, giving rise to a partial ventralization. In contrast, in the double *alk1/2* + *alk3/6* morphants, the whole ectoderm is converted into ventral ectoderm. LV, lateral view; VV, vegetal pole view; AV, animal pole view; FV, frontal view; V, ventral; D, dorsal.

To further characterize the requirements for Alk1/2 and Alk3/6 in D/V axis patterning, we performed a double knockdown. Our expectations were that the double knockdown of *alk3/6* and *alk1/2* would produce a phenotype roughly similar to the BMP2/4 loss-of-function phenotype. However, surprisingly, the morphology of the double knockdown embryos was very different from that of the *bmp2/4* knockdown. The *alk1/2* + *alk3/6* morphants were completely radialized and developed with a prominent proboscis in the animal pole region and with an ectopic ciliary band surrounding the vegetal pole region (Fig 2A, white and black arrowheads, respectively). These features are typical of the strongly ventralized phenotype observed in *nodal*-overexpressing or nickel-treated embryos (Fig 2A). Indeed, molecular analysis revealed that the double inhibition of Alk3/6 and Alk1/2 caused a massive ectopic expression of *nodal* and of its downstream target genes *chordin* and *foxA* in the presumptive ectoderm at the blastula stage, whereas it abolished the expression of the dorsal marker gene *hox7* (Fig 2B and 2C). Consistent with the extreme ventralization of the double *alk1/2* + *alk3/6* morphants, at the gastrula stage, expression of the ciliary band genes *foxG* and *onecut* was detected in a belt of cells surrounding the vegetal pole (black arrowheads in Fig 2B), a pattern typically observed in embryos ventralized by *nodal* overexpression (Fig 2C) [30]. Since these results suggest that signaling from these BMP receptors is required to restrict *nodal* expression, we tested if treatments with recombinant BMP2/4 can antagonize *nodal* expression. Indeed, treatments with increasing concentrations of recombinant BMP2/4 protein gradually antagonized *nodal* expression, with low concentrations first causing a typical Nodal loss-of-function phenotype and high concentrations resulting in dorsalization of the ectoderm (S2 Fig) [30].

Taken together, these results reveal that specification of the ventral territory is not independent of BMP signaling, as previously thought [25,30]. The results suggest instead that, in addition to specifying the dorsal region at the onset of gastrulation, signaling from the two BMP receptors Alk3/6 and Alk1/2 is critically required during or before blastula stages to restrict *nodal* expression to the ventral side. Importantly, the fact that the *bmp2/4* morphant phenotype is considerably weaker than the double *alk1/2* + *alk3/6* morphant phenotype strongly suggests that an unidentified signal acting through these BMP type I receptors is critically required, in addition to BMP2/4, for the correct specification of the D/V axis and for the normal restriction of *nodal* expression.

### The TGF-β Ligand Panda Cooperates with BMP2/4 to Restrict *nodal* Expression during D/V Patterning

We next attempted to identify the TGF-β ligand acting through Alk3/6 and Alk1/2 that cooperates with BMP2/4 and that restricts the early expression of *nodal* during D/V axis formation. In other species, BMP ligands of the BMP5/8 and anti-dorsalizing morphogenetic protein (ADMP) subfamilies have been shown to cooperate and to act redundantly with BMP2/4 factors during D/V patterning. For example, in *Xenopus*, while the single knockdown of either ADMP, BMP2, BMP4, or BMP7 resulted in partial central nervous system (CNS) expansion, the quadruple knockdown of BMP2,4,7 and ADMP caused full radialization and ubiquitous neural induction [31,32]. We therefore tested if in the sea urchin, like in *Xenopus*, members of the BMP5/8 and ADMP subfamilies of TGF-β ligands cooperate with BMP2/4 during D/V patterning.

**Fig 3:**
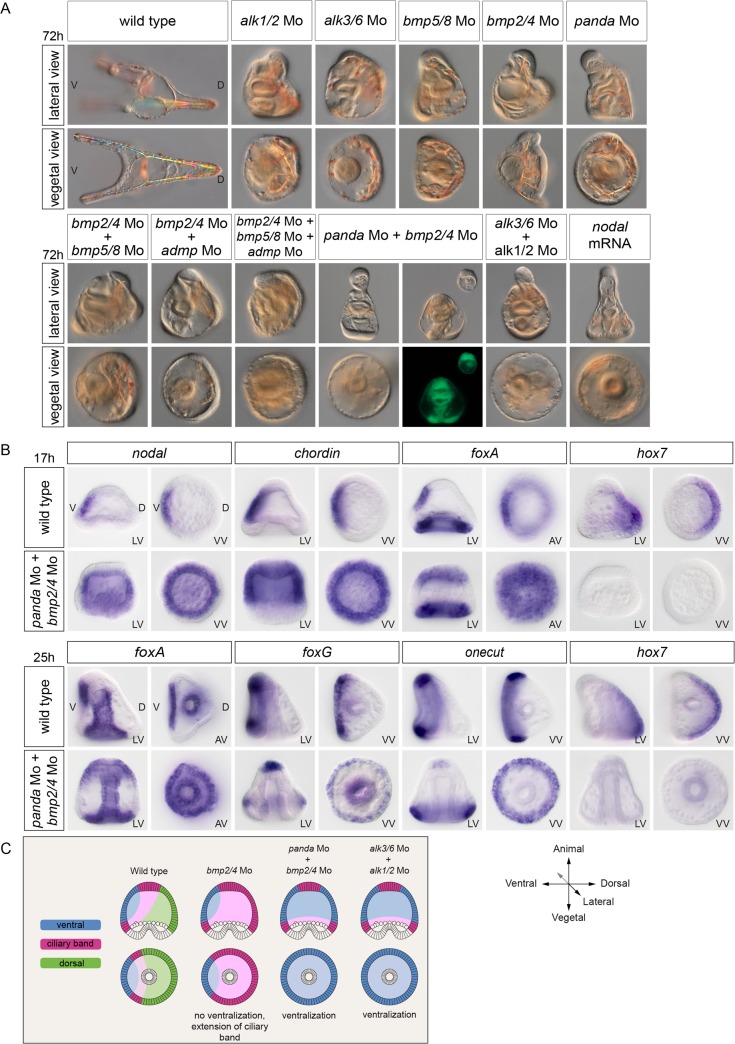
Panda is the TGF-β ligand that cooperates with BMP2/4 to restrict *nodal* during D/V patterning. (A) Simple inactivation of *alk1/2*, *alk3/6*, *bmp5/8*, *bmp2/4*, *panda*, double inactivation of *bmp5/8* +*bmp2/4* or of *bmp2/4* +*admp*, or triple inactivation of *bmp2/4*+ *bmp5/8* + *admp* affects D/V polarity to various extents but does not cause full ventralization of the embryo. In contrast, double inactivation of *panda* and *bmp2/4* causes an extreme ventralization of the embryo, mimicking the phenotype caused by *nodal* overexpression or by the double inactivation of *alk1/2* and *alk3/6*. The ventralized phenotype of the double *panda* + *bmp2/4* morphants is so strong that the proboscis in the animal pole frequently detaches from the rest of the embryo as a consequence of formation of a circular stomodeum. (B) In situ hybridization on controls and double *panda* + *bmp2/4* morphants at the blastula and gastrula stages with ventral, ciliary band, and dorsal marker genes. Simultaneous inactivation of *panda* and *bmp2/4* causes massive ectopic expression of *nodal*, suppresses dorsal marker gene expression, and restricts ciliary band markers to the vegetal pole, mimicking the effects of the double knockdown of Alk1/2 + Alk3/6. (C) Scheme describing the changes in fate maps caused by the single inactivation of *bmp2/4*, by the double inactivation of *bmp2/4* and *panda*, or by the double inactivation of *alk1/2* and *alk3/6*. LV, lateral view; VV, vegetal pole view; AV, animal pole view; V, ventral; D, dorsal.

The simple knockdown of BMP5/8 caused a phenotype much weaker than the phenotype caused by blocking BMP2/4. Surprisingly, the double knockdown of BMP2/4 and BMP5/8 only slightly increased the severity of the BMP2/4 morphant phenotype (Fig 3A and S3 Fig). Similarly, the double knockdown of BMP2/4 and ADMP did not cause a phenotype dramatically more severe than the BMP2/4 morphant phenotype. Even more surprising, the triple knockdown of BMP2/4, BMP5/8, and ADMP did not increase significantly the severity of the BMP2/4 morphant phenotype and did not result in ventralized embryos, suggesting that in the sea urchin, BMP5/8 and ADMP do not act redundantly with BMP2/4 to regulate the spatial restriction of Nodal (S3 Fig). We therefore extended our search for TGF-β ligands that would cooperate with BMP2/4 during D/V patterning to other members of the TGF-β superfamily. In addition to members of the BMP subfamily such as *bmp2/4*, *bmp5/8*, and *admp*, the sea urchin genome contains several genes encoding TGF-β ligands structurally related to Activins including TGF-β sensu stricto, Activin as well as SPU_018248, a less well-characterized gene related to Maverick from *Drosophila* that we renamed *panda (paracentrotus anti-nodal dorsal activity)* (see below) in this study [33]. Blocking Activin or TGF-β sensu stricto did not perturb establishment of the D/V axis, making unlikely the possibility that these factors cooperate with BMP2/4 to restrict *nodal* expression [34] (our unpublished results). In contrast, blocking translation of the TGF-β Panda strongly affected D/V polarity. While the triple knockdown of *bmp2/4*, *bmp5/8*, and *admp1* did not increase the severity of the *bmp2/4* morphant phenotype, in contrast, the double knockdown of *bmp2/4* and *panda* produced a very strong phenotype, indistinguishable from that of the double *alk1/2 + alk3/6* morphants. Strikingly, the ventralization induced by the double inactivation of *panda* and *bmp2/4* was so strong that it frequently led to scission of the embryos in two parts by formation of a circular stomodeum and separation of the animal pole-derived proboscis from the vegetal part of the larva that contained the gut (Fig 3A). Indeed, starting at early stages, the double *panda* + *bmp2/4* morphants displayed a massive ectopic expression of *nodal*, similar to that caused by the double inactivation of Alk1/2 and Alk3/6 (Fig 3B). The extent of this radialization was extremely pronounced, as evidenced by the radial expression of the other ventral markers *chordin* and *foxA* and of the ciliary band markers *onecut* and *foxG* as well as by the suppression of the dorsal marker *hox7* both at the blastula and late gastrula stages. The summary diagram of Fig 3C shows that, while inactivation of *bmp2/4* alone does not cause ventralization, in contrast, simultaneous inactivation of both *panda* and *bmp2/4*, like the double knockdown of *alk1/2* and *alk3/6*, causes unrestricted expression of *nodal* leading to strong ventralization.

These observations strongly support the view that Panda is the elusive factor that, together with BMP2/4, is required to antagonize Nodal signaling during D/V patterning of the embryo. Taken together, these results also suggest that, in addition to Lefty, the normal restriction of *nodal* expression during D/V patterning in the sea urchin embryo requires the activities of Panda and BMP2/4 possibly signaling through the two BMP type-I receptors, Alk3/6 and Alk1/2.

### Panda Is a Maternally Expressed TGF-β Ligand Required Early to Restrict *nodal* Expression

**Fig 4:**
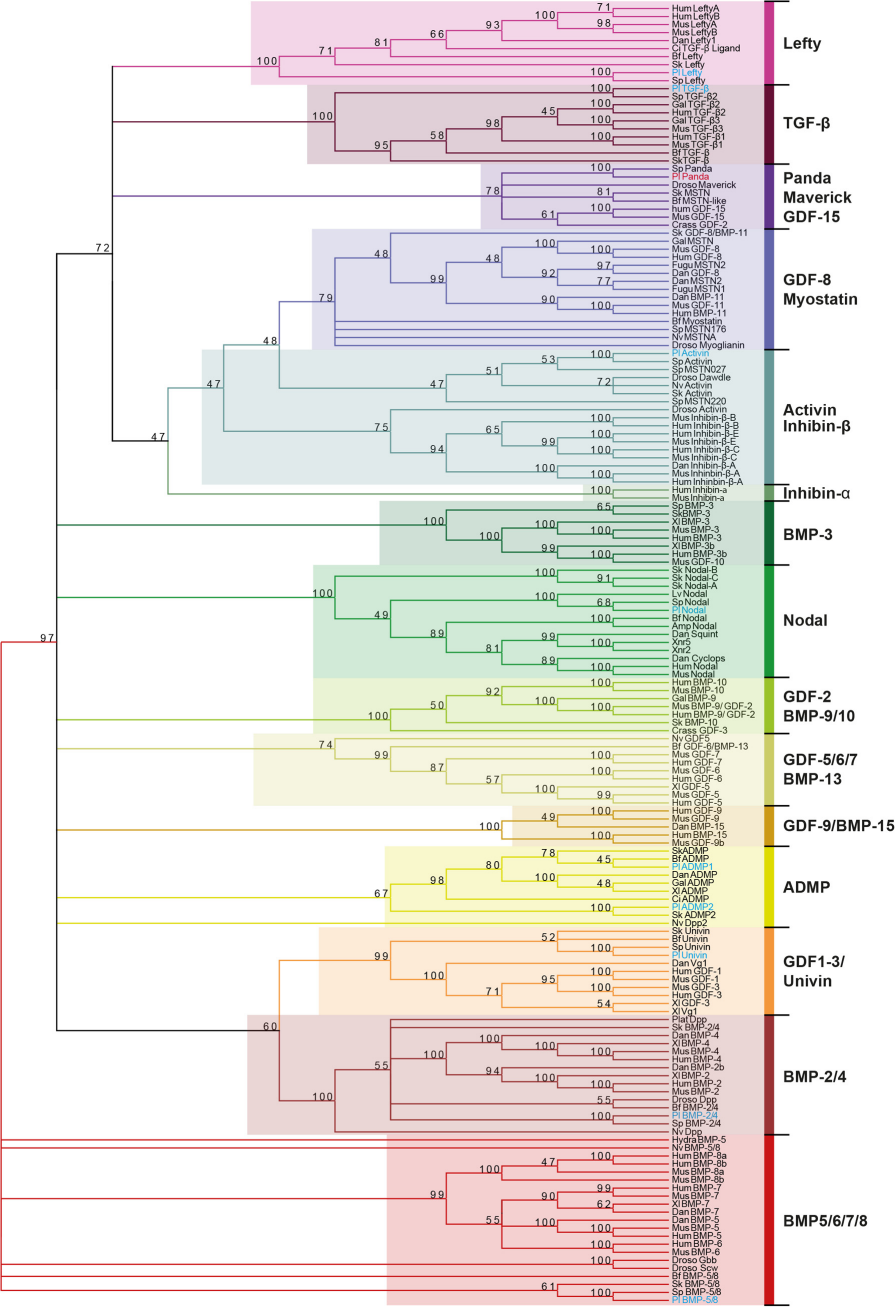
Panda belongs to a subfamily of TGF-β that includes *Drosophila* Maverick and vertebrate GDF15. Phylogenetic analysis of TGF-β ligands using the maximum likelihood method. The analysis was performed using the full-length proteins. Representative taxa from deuterostomes, protostomes, and cnidarians were used (see S1 Text for a list of these taxa). These 162 sequences were collected from diverse databases using the National Center for Biotechnology Information (NCBI) research tool (http://www.ncbi.nlm.nih.gov/). Full-length sequences were aligned using ClustalOmega with default parameters (http://www.ebi.ac.uk/clustalw/), and gap optimisation and obvious alignment error correction were made using Bioedit 7.0.5.3 (http://www.mbio.ncsu.edu/BioEdit/bioedit.html). The tree was calculated using the maximum likelihood method with PhyML with substitution model WAG (http://atgc.lirmm.fr/phyml/). A consensus tree with a 45% cutoff value was derived from 500 bootstrap analysis using Mega 3.1 (http://www.megasoftware.net/). Numbers above nodes represent a percentage of bootstrap values supporting this node. The original tree is presented in S5 Fig.

In a previous study, we had suggested that the TGF-β encoded by SPU_018248 is related to Maverick sequences from insects and to GDF2 sequences from *Crassostrea gigas*; however, this analysis failed to identify any deuterostome orthologue of this gene, and the evolutionary origin of this TGF-β remained unclear [33]. To clarify the evolutionary relationships between SPU_018248 and other TGF-β family members and to identify orthologous sequences of this gene in deuterostomes, we performed a novel phylogenetic analysis using a comprehensive set of TGF-β sequences from protostomes, deuterostomes, and cnidarians and including in the analysis the Maverick sequence from *Drosophila* and the GDF2 sequence from Molluscs as well as a large set of BMP family members from different organisms (Fig 4 and S4 Fig). This analysis confirmed that the sea urchin Panda sequence is phylogenetically related to *Drosophila* Maverick and "GDF2-like" sequence from *Crassostrea*. However, it further revealed that Panda and Maverick/GDF15-like factors belong neither to the GDF2/BMP9 family nor to any known subclass of canonical BMP ligands. In addition, this analysis identified GDF15 from vertebrates as well as two genes from hemichordates and cephalochordates (called *myostatin-like*) as additional deuterostome orthologues of Panda (see also S6 Fig). Consistent with these conclusions, Panda, Maverick, and GDF15 share with Inhibins beta chains, TGF-β, and Myostatins a pattern of nine cysteines in the ligand domain, a pattern that is not shared by any prototypical BMP ligand (see S5 Fig and the alignment provided in the supplementary information). Therefore, Panda, Maverick, and GDF15-like sequences define a distinct subclass of TGF-β ligands within a larger branch of the TGF-β superfamily that comprises Inhibins beta chains, Lefty factors, Myostatins, and TGF-β sensu stricto (see also [35]).

**Fig 5:**
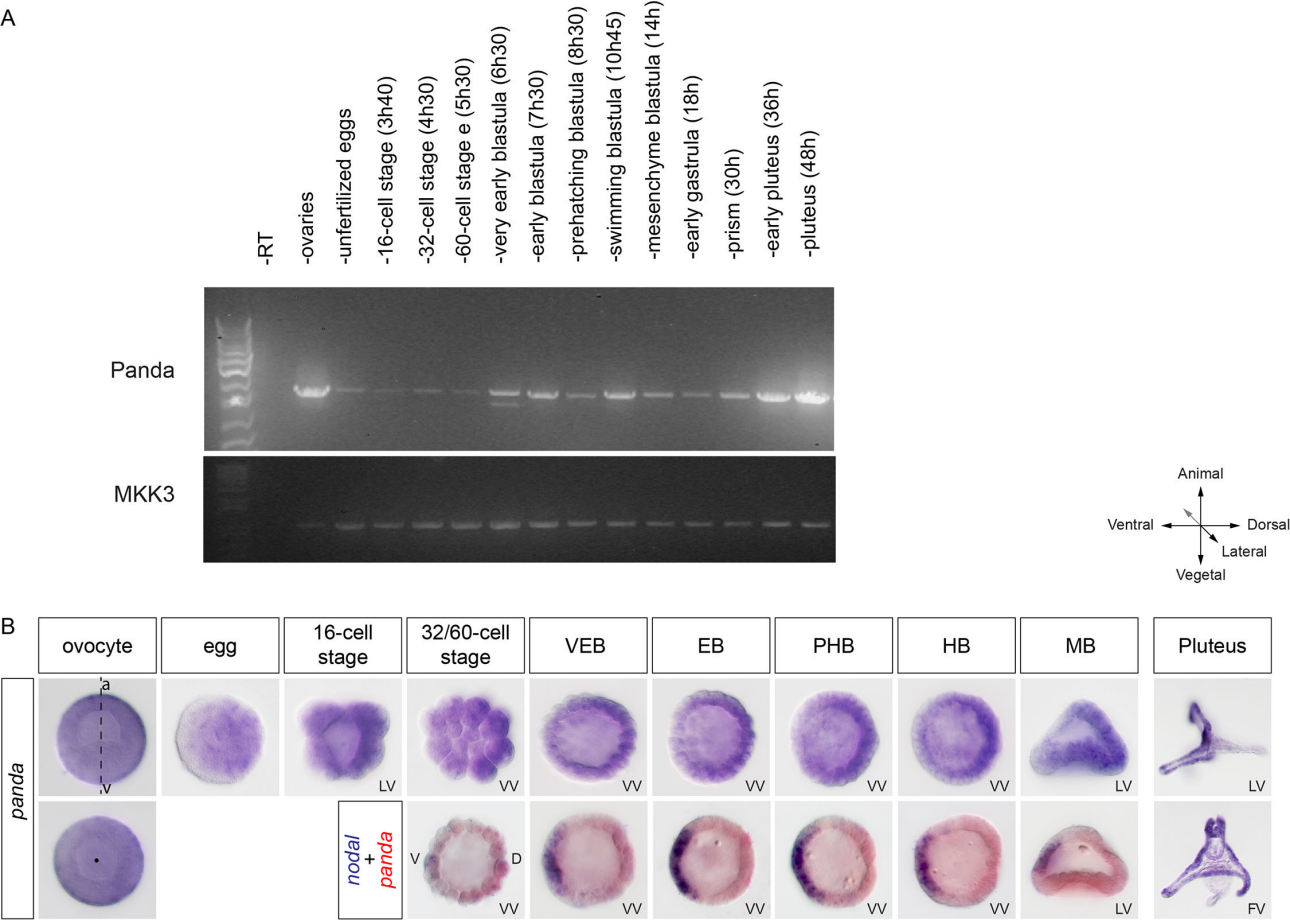
The TGF-β ligand Panda is expressed maternally in a D/V gradient. (A) RT-PCR analysis of Panda mRNA. An aliquot of the PCR reaction was run on a 1% agarose gel, and the gel was stained with Syber safe. Top panel, *panda* expression. Bottom panel, expression of *mkk3* used as a control. Panda is expressed in ovaries and unfertilized eggs as well as during the cleavage, blastula, gastrula, and pluteus stages. (B) Expression of *panda* mRNA analyzed by in situ hybridization. Whole mount in situ hybridizations with *panda* alone or with *panda* (red) and *nodal* (blue) probes. A gradient of maternal *panda* mRNA is detected in immature ovocytes and to a lesser extent in the unfertilized mature eggs, whereas during the cleavage and blastula stages, *panda* mRNA is detected in a shallow D/V gradient. VEB, very early blastula (about 120 cells); EB, early blastula (about 220 cells); PHB, prehatching blastula (about 300 cells); HB, hatching blastula (about 400 cells); MB, mesenchyme blastula; LV, lateral view; VV, vegetal view; V, ventral; D, dorsal; FV, frontal view.

Previous studies on sea urchin *maverick/panda* failed to detect expression of this gene by in situ hybridization [33], while by using an oligonucleotide microarray, a very weak expression was detected in 2 h zygotes and in 72 h pluteus larvae [36]. We reanalyzed the expression of *panda* by reverse transcription polymerase chain reaction (RT-PCR) and in situ hybridization and confirmed that transcripts of this gene are present predominantly in immature ovocytes, unfertilized eggs, and early embryos (Fig 5A and 5B and S9 Fig). Remarkably, a graded distribution of transcripts could be detected in immature ovocytes, in eggs, and during early stages, with one side of the embryo showing a slightly stronger staining than the other, reinforcing the idea that this factor plays an early role in D/V axis formation. Furthermore, double labeling with *nodal* revealed that the side with the highest concentration of mRNA was the dorsal side, opposite to the side of *nodal* expression, consistent with the idea that Panda is a factor that cooperates with BMP2/4 to restrict *nodal* expression (Fig 5B). Finally, starting at the prism stage, *panda* transcripts accumulated in the ciliary band territory, and strong expression was detected in individual cells within this territory.

**Fig 6:**
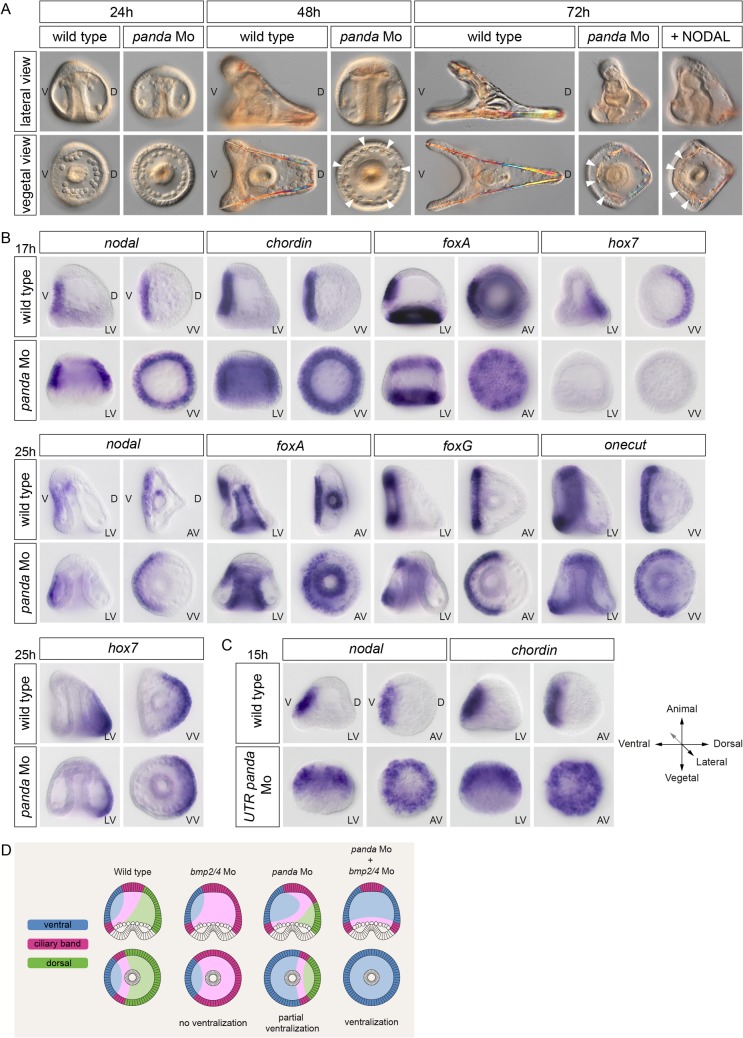
Panda plays a pivotal role during D/V axis formation. (A) Morphological phenotypes resulting from injection into the egg of antisense morpholino oligonucleotide targeting the translation start site of the *panda* transcript. Down-regulation of *panda* completely radializes the embryos during the first 48 h, but a partial recovery of D/V polarity occurs afterwards, as evidenced by the formation of a short dorsal apex. This phenotype is similar to that caused by treatments with recombinant Nodal protein at 1 μg/ml. (B) In situ hybridization on control embryos and *panda* morphants at the blastula and gastrula stages with ventral, ciliary band, and dorsal marker genes. Note the dramatic ectopic expression of *nodal*, *chordin*, and *foxA* at the blastula stage in *panda* morphants. At the late gastrula stage, despite their radialized morphology, *panda* morphants are patterned along the D/V axis, as evidenced by the restricted expression of *nodal*, *foxA*, *foxG*, *onecut*, and *hox7*. Note, however, the extended expression of these ventral markers compared to control embryos. (C) A second morpholino oligonucleotide targeting the 5' UTR of the *panda* transcript produces similar phenotypes and radializes the expression of *nodal* and *chordin*. (D) Scheme describing the changes in fate maps caused by the single inactivation of *panda* or *bmp2/4* or by the double inactivation of *panda* and *bmp2/4*. LV, lateral view; VV, vegetal pole view; AV, animal pole view; V, ventral; D, dorsal.

To further characterize the role of *panda* during D/V axis formation, we injected two different antisense morpholino oligonucleotides targeting either the translation start site or the 5' UTR region of the transcript. Injecting these two different morpholinos gave rise to similar and remarkable phenotypes (Fig 6). While at the late gastrula stage, control embryos had started to flatten on the presumptive ventral side and had formed two PMC clusters on each side of the archenteron, the *panda* morphants were completely radialized, and the PMCs remained arranged into a ring around the archenteron (Fig 6A). Similarly at 48 hpf, when control embryos had developed into elongated pluteus larvae, *panda* morphants had conserved a radially symmetrical morphology and contained ectopic spicules rudiments (arrowheads in Fig 6A). Surprisingly, at 72 h, these embryos had partially recovered a D/V polarity as indicated by the bending of the archenteron towards the presumptive ventral ectoderm, the opening of the stomodeum, and the preferential elongation of spicules on the presumptive dorsal side (Fig 6A). Indeed, molecular analysis revealed that in most of the embryos (*n* > 300), knocking down *panda* with either the ATG morpholino (Fig 6B) or the UTR morpholino (Fig 6C) caused a strong ventralization accompanied with massive ectopic expression of *nodal* and *chordin*, which were expressed throughout most of the ectoderm at the mesenchyme blastula stage, and a concomitant loss of the dorsal marker gene *hox7*. At the late gastrula/prism stage (30 hpf), *panda* morphants remained ventralized, as evidenced by the expanded expression of ventral marker genes such as *nodal*, *foxA*, and *foxG* compared to control embryos, occupying about one-half of the embryo. However, consistent with the progressive recovery of D/V polarity observed in live embryos, the expression of *hox7* in the dorsal region and of the ciliary band marker *onecut* at the late gastrula stage indicated that dorsal and ciliary band fates were allocated in *panda* morphants by the end of gastrulation (Fig 6B). Therefore, although the morphology of *panda* morphants is radially symmetrical at late gastrula stage, molecular analysis reveals that these embryos are nevertheless patterned along the D/V axis and that radialization is caused by a marked expansion of ventral cell fates. Taken together, these results suggest that Panda function is required early to restrict *nodal* expression. In the absence of Panda, ventral fates are expanded at the expense of dorsal fates, but this ventralization is most severe during the blastula and gastrula stages, the embryos progressively recovering, to some extent, a D/V polarity after 48 h (Fig 6D).

### Panda, Alk1/2, and Alk3/6 Are Required Early to Restrict *nodal* Expression

**Fig 7:**
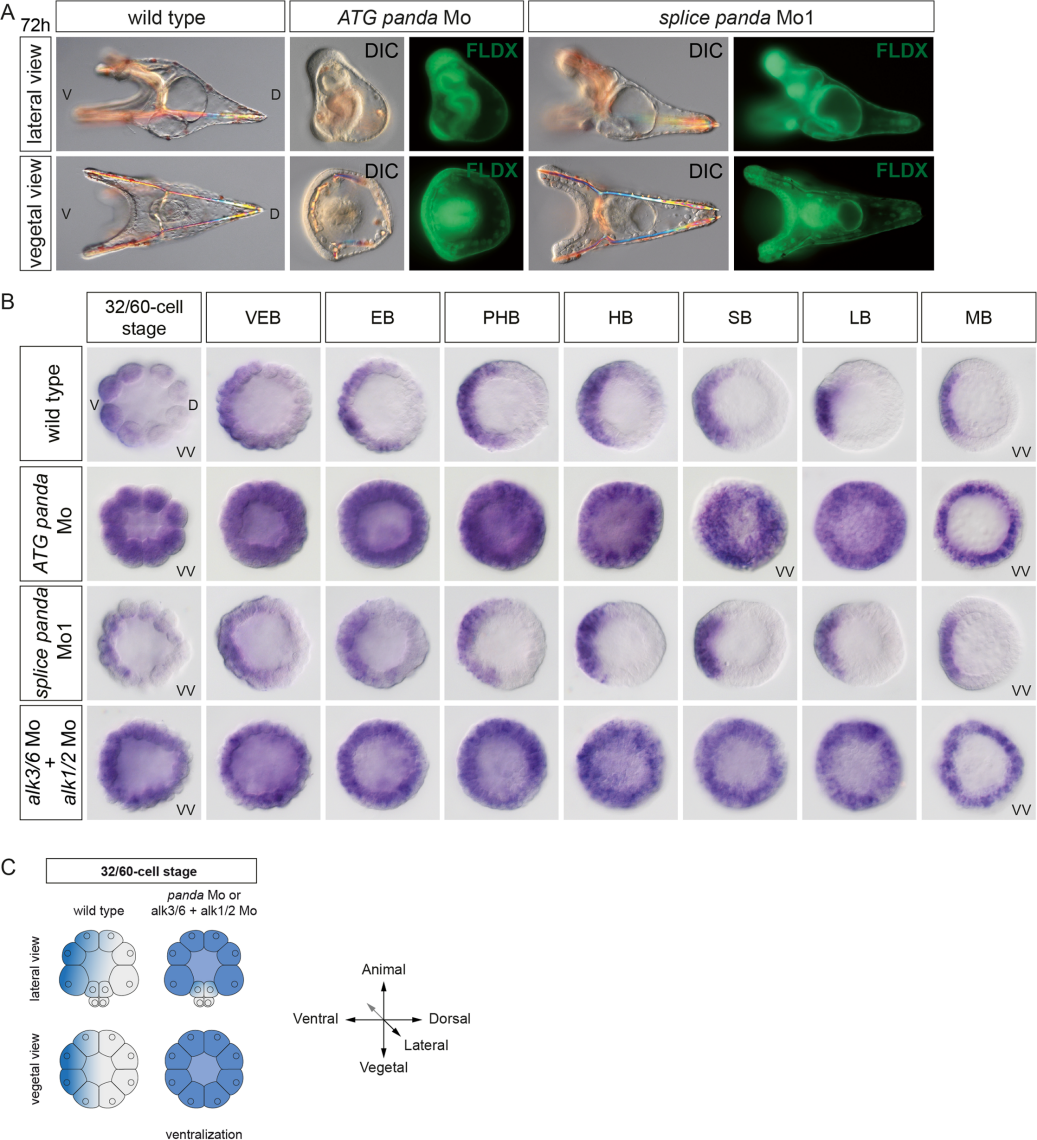
Maternal but not zygotic Panda function is required for the spatial restriction of *nodal* expression. (A) Injection of a morpholino oligonucleotide targeting the translation start site of *panda* mRNA, but not of a morpholino targeting a splice junction, disrupts the establishment of D/V polarity. The lineage tracer Fluoresceinated Lysine-Fixable Dextran (FLDX) was coinjected with the morpholino. (B) In situ hybridizations against the *nodal* transcript at early stages. In the absence of maternal, but not of zygotic, Panda, a massive ectopic expression of *nodal* is observed starting at the 60-cell stage. Note that *nodal* expression remains radially expressed up to the mesenchyme blastula stage. Massive and early ectopic expression of *nodal* is also observed in the double *alk1/2*+*alk3/6* morphants. VEB, very early blastula (about 120 cells); EB, early blastula (about 220 cells); PHB, prehatching blastula (about 300 cells); HB, hatching blastula (about 400 cells); SB, swimming blastula; LB, late blastula; MB, mesenchyme blastula. (C) Scheme summarizing the perturbations of *nodal* expression caused by blocking Panda or Alk1/2+Alk3/6. VV, vegetal pole view; V, ventral; D, dorsal.

To determine when Panda, Alk1/2, and Alk3/6 functions are required to restrict *nodal* expression, we performed a time-course experiment. We compared *nodal* expression at successive developmental stages, from cleavage to mesenchyme blastula, in control embryos and in embryos injected with either the morpholino oligonucleotide targeting the ATG of *panda* mRNA or with a morpholino oligonucleotide targeting a splice junction of the *panda* gene or with a mixture of *alk1/2* and *alk3/6* morpholino (Fig 7A–7C). Strikingly, in embryos injected with the morpholino targeting the translation start site of *panda* mRNA, presumed to block both maternal and zygotic *panda* transcripts, or with a combination of the *alk1/2* and *alk3/6* morpholinos, *nodal* expression was never restricted and remained radialized at all stages analyzed (Fig 7B and 7C). In contrast, *nodal* expression was largely normal in embryos injected with the morpholino targeting the splice junction of *panda* (Fig 7B), and blocking zygotic *panda* function did not noticeably perturb development of the embryos (Fig 7A and S7 Fig). RT-PCR analysis indicated that this splice-blocking morpholino reduced the level of the mature panda transcript by more than 90% at the pluteus stage (S7 Fig). This analysis reveals that the function of maternal Panda, but not of zygotic Panda, and the activities of Alk1/2 and Alk3/6 are required very early to restrict *nodal* expression to the ventral side.

### Local Overexpression of *panda*, Like Inhibition of Nodal Signaling, Efficiently Orients the D/V Axis

**Fig 8:**
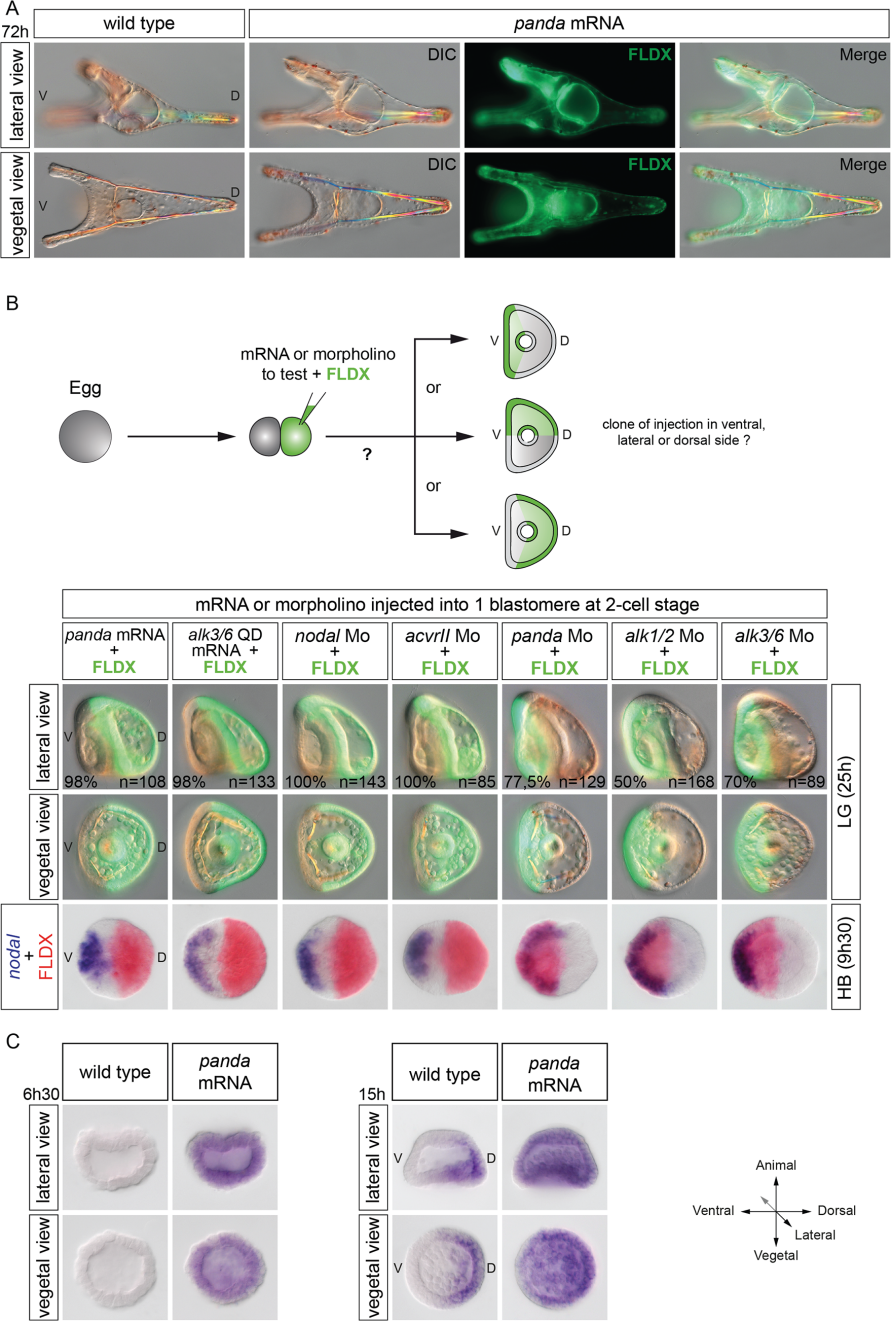
Panda activity is required locally to orient the D/V axis. (A) Global overexpression of *panda* at the one-cell stage does not perturb establishment of the D/V axis. Normal morphology of pluteus larvae developing after injection with *panda* mRNA at 1,000 μg/ml. (B) Effects of local overexpression or down-regulation of various components of the Nodal and BMP pathway on the orientation of the D/V axis. Injection of *panda* (1,000 μg/ml) or of the activated form of *alk3/6* (*alk3/6Q230D)* mRNA (200 μg/ml) into one blastomere at the two-cell stage imposes a dorsal identity to the progeny of the injected cell in nearly 100% of the injected embryos. Local down-regulation of *nodal* or *acvrII* also imposes a dorsal identity. Conversely, down-regulation of *panda* or *alk3/6* mRNA, and to a lesser extent of *alk1/2*, strongly biases the orientation of the D/V axis and promotes ventral fates. V, ventral; D, dorsal. The same result was also obtained by local inhibition of Nodal signaling after injection into one blastomere at the two-cell stage of a morpholino oligonucleotide targeting either the *nodal* transcript or the type II Nodal receptor *acvrII*. Consistent with this strong effect on the orientation of the D/V axis, in all embryos injected with *panda* or *alk3/6QD* mRNAs or with the *acvrII* or *nodal* morpholinos, at the blastula stage, *nodal* was expressed in a sector of the embryo located on the opposite side of the clone of injected cells (Fig 8B). (C) Expression of *tbx2/3* at the early blastula or mesenchyme blastula stages in embryos injected with *panda* mRNA. Overexpression of *panda* precociously and ubiquitously activates *tbx2/*3 at the early blastula stage. Note, however, that expression of *tbx2/3* becomes polarized along the D/V axis at the mesenchyme blastula stage.

The results presented so far indicate that Panda is expressed in a broad D/V gradient and that, like Lefty, Panda is critically required for the correct spatial restriction of *nodal* to the ventral side during early stages. We then tested if overexpression of Panda, like overexpression of Lefty, efficiently blocks Nodal signaling. Surprisingly, overexpression of *panda* in the egg did not perturb establishment of the D/V axis, and the *panda*-overexpressing embryos developed into normal pluteus larvae (Fig 8A). This suggested that unlike Lefty, Panda alone is not capable of suppressing Nodal signaling when overexpressed. We then reasoned that rather than inhibiting Nodal signaling, the function of Panda may instead be to bias early Nodal signaling, perhaps by simply attenuating Nodal signaling on the dorsal side. If this were the case, then local overexpression of *panda* should efficiently orient the D/V axis. To test if local overexpression of *panda* is capable of biasing the orientation of the D/V axis, embryos at the two-cell stage were injected into one blastomere with *panda* mRNA together with a lineage tracer, and at the prism stage, the position of the clone of injected cells was recorded (Fig 8B). Strikingly, in almost 100% of the embryos injected with *panda* mRNA, the boundaries of the clone precisely coincided with the dorsal part of the embryo. Local overexpression of a constitutively active version of Alk3/6 (Alk3/6QD) or Alk1/2 (ALK1/2Q/D) mimicked the effects of local overexpression of *panda*, efficiently orienting the D/V axis in all the injected embryos (Fig 8B, Table 1).

**Table 1:** Ability of various components of the Nodal or BMP pathways to orient the D/V axis following misexpression into one blastomere at the two-cell stage.

	Localization of injection clone			*n* =
	ventral (%)	lateral (%)	dorsal (%)	
Fluorescent dextran	32	36	32	109
*panda* mRNA	1	1	98	108
*alk36QD* mRNA	0.5	1.5	98	133
*alk1/2QD* mRNA	0	0	100	37
*nodal* Mo	0	0	100	143
*ACVR2* Mo	0	0	100	85
*panda* Mo	77.5	16	8	129
*alk1/2* Mo	50	38	12	168
*alk3/6* Mo	70	21	9	89
*alk1/2* Mo + *alk3/6* Mo	83	10	7	48
*bmp2/4* Mo	37	30	33	126

Mo, morpholino antisense oligonucleotide.

Finally, we tested if overexpression of *panda* promotes expression of dorsal marker genes. We analyzed the expression of *tbx2/3*, the earliest zygotic expressed in all three germ layers in the presumptive dorsal region and that is thought to be induced by low levels of BMP signaling [24,25]. Overexpression of *panda* induced a moderate ectopic expression of *tbx2/3* in all three germ layers, suggesting that *panda*, like *bmp2/4*, can activate BMP target genes requiring a low level of BMP signaling (Fig 8C) [24,25].

We next tested if removing the function of *panda* from part of the early embryo is also sufficient to orient the D/V axis (Fig 8B and Table 1). Indeed, injecting the *panda* morpholino randomly into one blastomere at the two-cell stage significantly biased the orientation of the D/V axis, most embryos (77.5%) showing a clone of fluorescently labeled cells in the ventral region. Similarly, injection of *alk3/6* morpholino into one blastomere at the two-cell stage efficiently (70%) oriented the D/V axis, supporting the idea that Alk3/6 is involved in the early steps of D/V axis specification. Injection of the *alk1/2* morpholino also significantly biased the orientation of the D/V axis, imposing a ventral identity to the clone in about 50% of the injected embryos (Fig 8B). In contrast, injection of the *bmp2/4* morpholino into one blastomere did not significantly orient the D/V axis, 37% of the injected embryos displaying a clone of injected cells on the ventral side, further suggesting that *bmp2/4* is not involved in the early steps of axis specification (Table 1).

In summary, these results show that while manipulating the levels of BMP2/4 does not appear to have a strong effect on the orientation of the D/V axis, in contrast, up-regulating or down-regulating the levels of Panda or Alk3/6, and to a lesser extent of Alk1/2, in part of the early embryo strongly impacts on the orientation of the D/V axis, partially mimicking manipulations of the levels of Nodal signaling.

### Spatially Restricted Panda Signaling Specifies the D/V Axis

**Fig 9:**
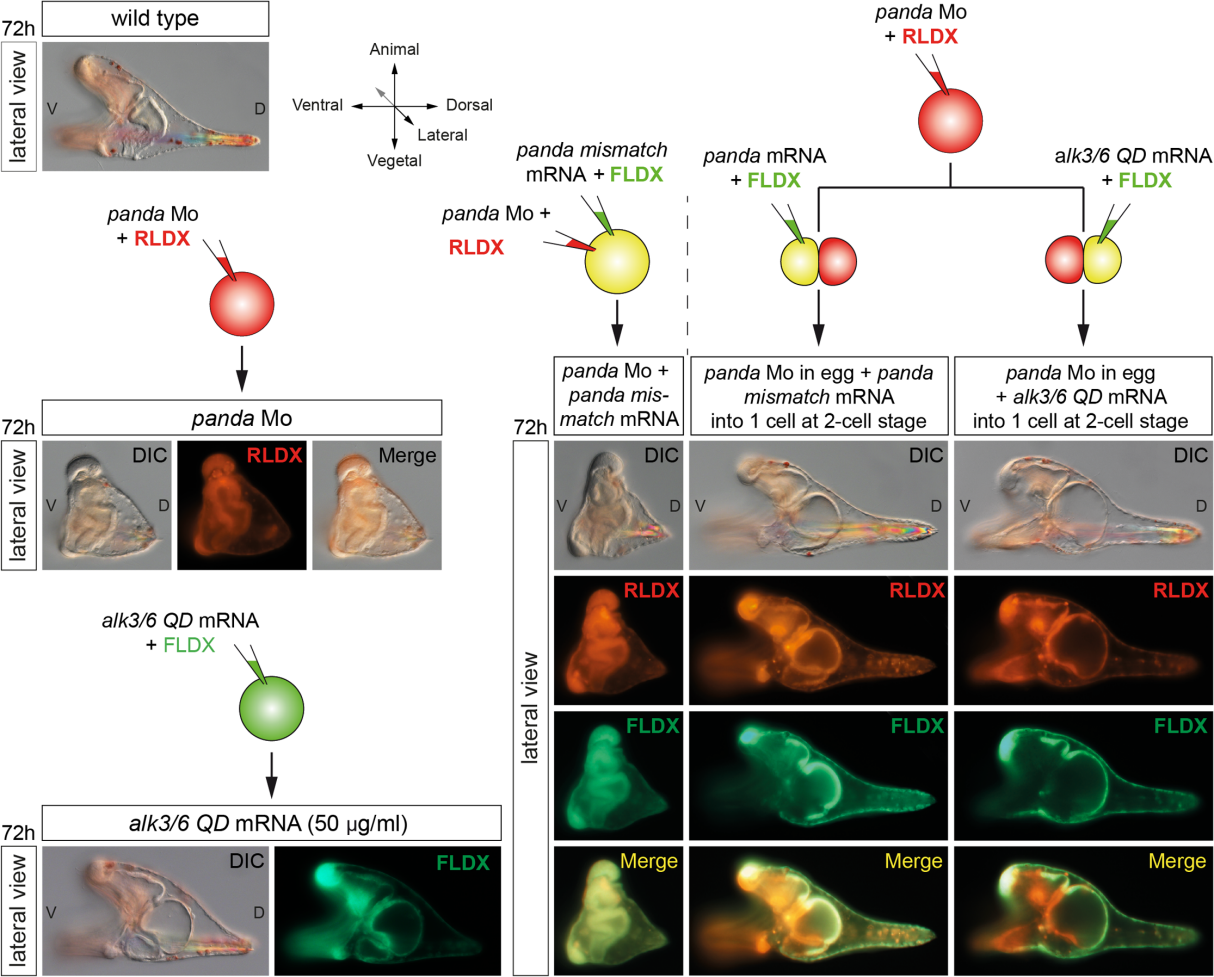
Panda activity has to be provided locally to efficiently rescue *panda* morphants. Differential interference contrast (DIC) and fluorescence images of embryos injected with a *panda* morpholino into the egg and then with *panda* mRNA either into the egg or into one blastomere at the two-cell stage. While providing Panda activity into the egg does not rescue D/V polarity, injection of *panda* mRNA or of low doses (50 μg/ml) of mRNA encoding the activated form of Alk3/6 (Alk3/6QD) into one blastomere at the two-cell stage fully rescues D/V polarity of *panda* morphants. LV, lateral view; V, ventral; D, dorsal.

In the course of our functional analysis of *panda*, we tried to rescue the defects of D/V patterning and the spatial restriction of *nodal* expression of *panda* morphants, by injecting a synthetic *panda* mRNA lacking the sequence targeted by the morpholino. Surprisingly, injection into the egg of a synthetic *panda* mRNA failed to rescue the severe defects of D/V polarity caused by injection of the *panda* morpholino (Fig 9). All the embryos derived from double injection of *panda* morpholino and *panda* mRNA at the one-cell stage developed with a phenotype indistinguishable from the *panda* loss-of-function phenotype. Since Panda is required to restrict *nodal* expression and since the endogenous *panda* mRNA is enriched on the presumptive dorsal side, we reasoned that maybe Panda activity had to be provided locally in order to mimic the distribution of endogenous *panda* mRNA and to rescue D/V polarity of *panda* morphants. Indeed, while injection of *panda* mRNA into the egg was inefficient to rescue the D/V axis, injection of *panda* mRNA into one blastomere completely rescued D/V polarity of embryos previously injected with the *panda* morpholino, all the embryos developing into perfectly normal pluteus larvae with the dorsal side corresponding to the *panda*-expressing clone (Fig 9). This experiment demonstrates that the activity of exogenous Panda has to be spatially restricted to rescue the lack of maternal Panda function, consistent with the idea that the activity of endogenous Panda is spatially restricted in the early embryo. Similarly, injection into one blastomere of *alk3/6QD* mRNA at low doses that do not dorsalize completely rescued D/V polarity of embryos previously injected with *panda* morpholino, consistent with previous results showing that local misexpression of an activated form of Alk3/6 is sufficient to antagonize *nodal* expression and to orient the D/V axis (Fig 8B).

### Panda Function Requires the BMP Type I Receptors Alk1/2 and Alk3/6 but Overexpression of Panda Does Not Promote Directly Phosphorylation of Smad1/5/8

**Fig 10:**
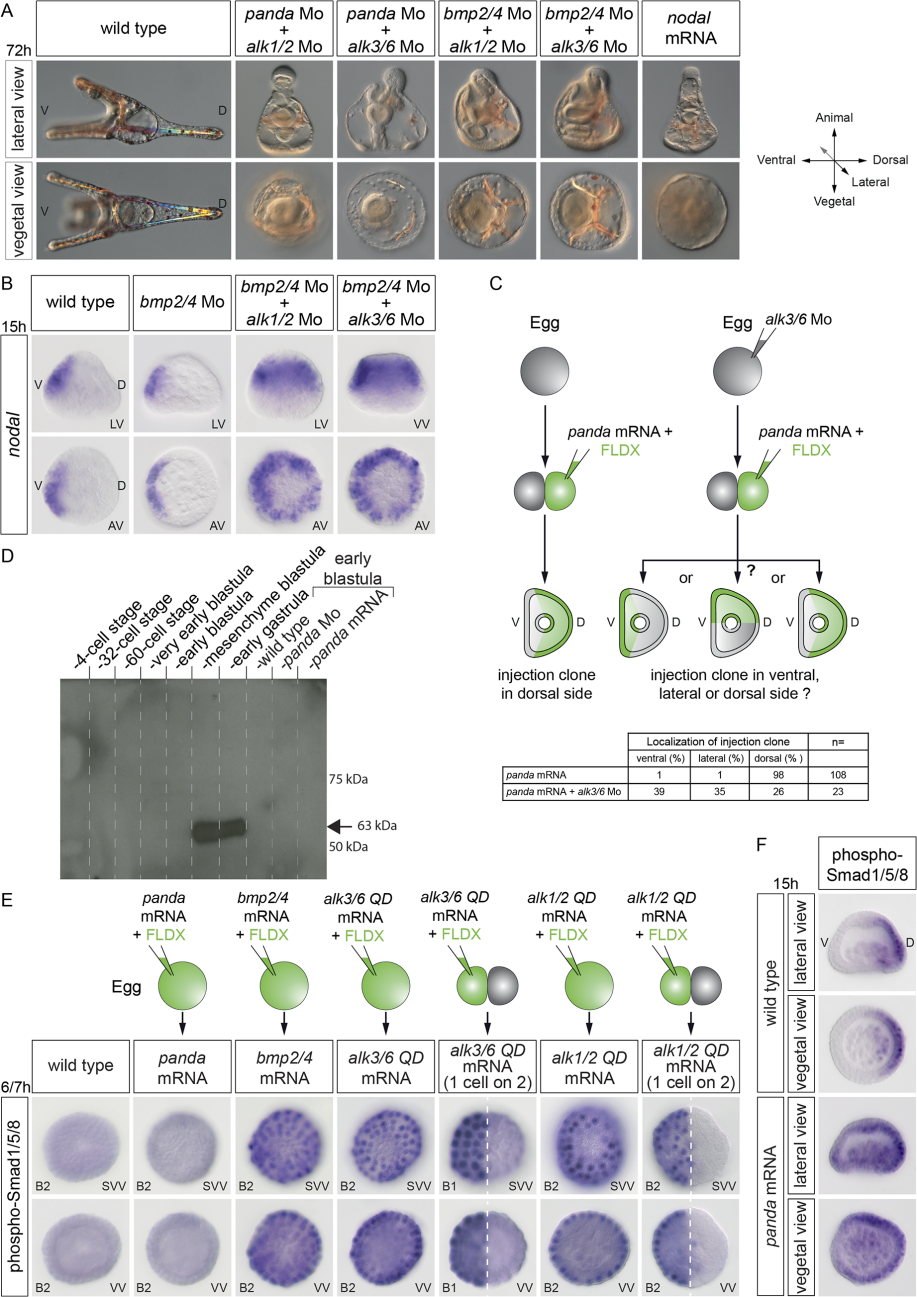
Panda, like BMP2/4, requires Alk1/2 and Alk3/6 to pattern the D/V axis, but Panda, unlike BMP2/4, does not appear to activate phosphorylation of Smad1/5/8. (A) Coinjection of the *panda* and *alk1/2* morpholinos or of the *panda* and *alk3/6* morpholinos causes a strong ventralization, as does the double inactivation of *bmp2/4* + *alk1/2* or of *bmp2/4* and *alk3/6*. (B) While the simple knockdown of *bmp2/4* is not sufficient to cause ectopic expression of *nodal*, the double knockdown of *bmp2/4* + *alk1/2* or *bmp2/4* + *alk3/6* causes massive ectopic expression of *nodal*. (C) Panda requires Alk3/6 to orient the D/V axis when misexpressed. Local overexpression of *panda* orients the D/V axis in nearly 100% of the injected embryos. However, if the eggs are first injected with the *alk3/6* morpholino, panda is no longer able to orient the D/V axis. (D) Western blot of phospho-Smad1/5/8 in control embryos at the indicated stages or in embryos overexpressing *panda* or injected with a *panda* morpholino. Note that phosphoSmad1/5/8 is undetectable before the late blastula stage and that overexpression of *panda* does not appear to cause phosphorylation of Smad1/5/8. B1, very early blastula; B2, early blastula. (E) Phospho-Smad1/5/8 immunostaining at very early and early blastula stages or mesenchyme blastula stages in control embryos and in embryos overexpressing *panda*, *bmp2/4*, *alk3/6QD* mRNA, or *alk1/2QD*. The highly sensitive alkaline phosphatase-based detection of pSmad1/5/8 does not allow detection of Smad1/5/8 signaling at early stages in control embryos. In contrast, following overexpression of *bmp2/4*, *alk3/6QD*, or *alk1/2QD* into the egg or into one blastomere at the two-cell stage, strong nuclear phosphoSmad1/5/8 immunostaining is easily detected at early blastula. This pSmad1/5/8 immunoreactivity is not detected following injection of *panda* mRNA. Nevertheless, overexpression of *panda* induces weak ectopic Smad1/5/8 signaling at mesenchyme blastula. (F) Overexpression of *panda* expands Smad1/5/8 signaling at the mesenchyme blastula stage. pSmad1/5/8 is normally restricted to the dorsal ectoderm and dorsal PMCs at mesenchyme blastula. Overexpression of *panda* expands the territory in which pSmad1/5/8 is detected toward the ventral side. LV, lateral view; VV, vegetal view; SVV, surface and vegetal view; V, ventral; D, dorsal.

The finding that knocking down Panda causes a phenotype similar to that caused by knocking down the two BMP type I receptors Alk1/2 and Alk3/6, leading to early ectopic expression of *nodal*, and the fact that local expression of *alk3/6QD* efficiently rescues D/V polarity in *panda* morphants indicated that Panda most likely uses Alk1/2 and Alk3/6 to signal. To further address the question of the specificity of the ligands regarding the receptors, we used an assay based on the double knockdown of Panda or BMP2/4 and Alk1/2 or Alk3/6 receptors (Fig 10).

Double inactivation of *panda* and *alk1/2* or of *panda* and *alk3/6* caused a strong ventralization similar to that caused by the double inactivation of *panda* and *bmp2/4*, consistent with the idea that the activities of Alk1/2 and Alk3/6 are both required to transduce BMP2/4 signals (Fig 10A). Similarly, the double inactivation of *bmp2/4* and *alk1/2* or of *bmp2/4* and *alk3/6* produced a strong ventralization, suggesting that the activities of Alk1/2 and Alk3/6 are both required to transduce Panda signals, although the phenotype was slightly less severe than that resulting from the double knockdown of *panda* and *bmp2/4* (Fig 10A and 10B).

To test directly the hypothesis that Panda requires the BMP type I receptors to orient the D/V axis, we used the axis induction assay. We first injected the *alk3/6* morpholino into the egg, and then, at the two-cell stage, we injected *panda* mRNA into one blastomere. While *panda* mRNA efficiently oriented the D/V axis when injected alone, previous injection of the *alk3/6* morpholino into the egg abolished the ability of *panda* mRNA to orient the D/V axis, suggesting that the Alk3/6 receptor is required for the activity of Panda (Fig 10C).

Taken together, the results presented above strongly suggest that Panda requires the activity of the BMP type I receptors to orient the D/V axis; however, they do not answer the question of what signal transduction pathway is activated by this ligand. Since the axis-inducing activity of Panda requires the BMP type I receptor Alk3/6 and since manipulating the levels of this BMP type I receptor largely mimicked the effects of manipulating the levels of Panda, we expected that Panda, acting through Alk3/6 and Alk1/2, would activate canonical BMP signaling and Smad1/5/8 phosphorylation. However, intriguingly, previous studies failed to detect phospho-Smad1/5/8 before the hatching blastula stage using western blotting [24,25], suggesting that previous detection methods were not sensitive enough or that Panda may not activate canonical Smad signaling. We therefore attempted to detect endogenous phospho-Smad1/5/8 during the cleavage/early blastula period using an optimized western blotting assay. We were able to detect very strong phosphorylation of endogenous Smad1/5/8 at mesenchyme blastula stages (Fig 10D). However, endogenous phospho-Smad1/5/8 remained below the level of detection during cleavage stages, and overexpression of *panda* did not detectably increase the level of phosphorylated Smad1/5/8 at early stages. We also tried to detect phospho-Smad1/5/8 during cleavage/early blastula by using a sensitive immunostaining protocol. Fixed embryos were incubated with the anti-phospho-Smad1/5/8 antibody and then with a secondary antibody coupled to alkaline phosphatase (Fig 10E). While overexpression of *bmp2/4* or of an activated form of *alk3/6* or of *alk1/2* induced robust and very strong phosphorylation of Smad1/5/8 starting during cleavage stages, overexpression of *panda* did not cause any detectable phosphorylation of Smad1/5/8 at these early stages (Fig 10E). However, intriguingly, at mesenchyme blastula, we consistently observed expanded phospho-Smad1/5/8 signals in most embryos overexpressing *panda* (Fig 10F), consistent with the observed ectopic expression of *tbx2/3* (Fig 8C). However, pSmad1/5/8 signaling remained strongly polarized along the D/V axis, consistent with the apparent inability of *panda* to completely dorsalize embryos.

Taken together, these results suggest that *panda* may not directly activate phosphorylation of Smad1/5/8 and that the expanded pSmad1/5/8 signals may result from Panda antagonizing Nodal signaling and/or promoting BMP2/4 signaling [37].

In conclusion, the results presented in this study show that in addition to Lefty, the spatial restriction of *nodal* expression critically requires the activity of the maternal TGF-β ligand Panda. Panda is required very early and locally for the spatial restriction of *nodal* expression and is sufficient to orient the axis when locally overexpressed. Taken together, these properties strongly suggest that Panda may act as a maternal determinant of D/V axis formation in the sea urchin embryo.

## Discussion

An important and still largely unanswered question in developmental biology is how embryonic axes emerge in highly regulative and radially symmetrical embryos such as in mammals. Does formation of the primary and secondary axes depend entirely on cell interactions and reaction-diffusion mechanisms in the zygote, as suggested by the large developmental plasticity of the early blastomeres, or does it rely in part on maternal cues deposited in the egg? The process of D/V axis formation in the sea urchin embryo provides an interesting system to address this question. The D/V axis of the sea urchin embryo is thought to be specified largely in the absence of maternal determinants, as evidenced by the conspicuous developmental plasticity of the early blastomeres, and to rely instead on an asymmetry of the expression of the zygotic gene *nodal* established by a reaction-diffusion mechanism with its antagonist Lefty. Yet, are the concepts of maternal determination of axis formation and regulative development necessarily mutually exclusive?

In this study, we uncovered a very important and early function for a maternally expressed TGF-β ligand in the orientation of the D/V axis of the zygote through the spatial regulation of *nodal* expression. A key observation that was at the basis of this work was the finding that double inactivation of Alk1/2 and Alk3/6 produced an extreme radialization due to the unrestricted expression of *nodal*. Since this phenotype was much stronger than the *bmp2/4* morphant phenotype, and since the effects of abolishing BMP signaling on *nodal* expression could be observed well before the onset of *bmp2/4* expression, the inescapable conclusion was that another TGF-β ligand acting through these two TGF-β receptors was cooperating with BMP2/4 during D/V axis formation. Using a double morpholino injection assay, we identified this factor as Panda, a TGF-β ligand related to Inhibins, TGF-β and Lefty factors. These findings strongly impact on the current models of D/V axis formation since they reveal that the spatial restriction of *nodal* expression critically requires a maternally provided spatial cue. In addition, by showing that the graded localization of a maternal RNA provides a blueprint of the D/V axis in the highly regulative sea urchin embryo, they also provide additional support to the idea that the concept of maternal determination of axis specification and developmental plasticity are not necessarily exclusive. Finally, our findings that the orientation of *nodal* expression in the early embryo is negatively controlled by the spatially restricted activity of a TGF-β ligand that requires BMP type I receptors highlight the crucial role played by the antagonism between Nodal and BMP signaling in axis specification and suggest that this antagonism may represent an ancestral way to specify the axes during development.

### The Spatially Restricted Activity of a Maternal TGF-β Ligand as the Initial Asymmetry That Specifies the D/V Axis of the Sea Urchin Embryo

The current prevailing model postulates that redox gradients generated by mitochondria asymmetrically distributed in the egg regulate the activity of redox-sensitive transcription factors that control the initial asymmetry of *nodal* expression [21–23,26]. However, although very attractive, the hypothesis that mitochondrial redox gradients drive *nodal* expression is not strongly supported by the extensive experimental work that has addressed this question.

Here we provided several lines of evidence demonstrating that the maternally expressed TGF-β ligand Panda acts as an early and central player in the establishment of the D/V axis. First, we showed that the function of Panda is required very early to restrict *nodal* expression. Second, we showed that *panda* mRNA is expressed in a broad gradient in the early embryo and that the activity of Panda is spatially restricted. Third, we showed that overexpression of *panda* promotes the overexpression of *tbx2/3*, the earliest zygotic dorsal gene marker. Finally, we showed that local misexpression of *panda* mRNA or local inhibition of *panda* very efficiently orients the D/V axis. The broad distribution of Panda mRNA raises the possibility that some other localized factor may be required in the normal embryo for imposing D/V polarity on Panda function. The fact that only local overexpression, and not global overexpression, of Panda rescues the D/V axis of Panda morphants strongly suggests that this is probably not the case, since if another localized factor was playing the role of a maternal determinant, then injection of *panda* mRNA into the egg would rescue the D/V axis of *panda* morphants. Therefore, Panda is, to our knowledge, the first signaling factor whose activity is spatially restricted in the embryo and that is both necessary and sufficient to efficiently orient the D/V axis upstream of *nodal* expression. How can we reconcile the roles of Panda as a maternal signal that orients the D/V axis with the wealth of data correlating redox gradients and the asymmetric distribution of mitochondria with the secondary axis? One possible mechanism that would reconcile the two bodies of evidence is that formation of the gradient of *panda* mRNA may be dependent on the activity or the distribution of mitochondria. Alternatively, redox gradients could differentially affect the stability/activity of Panda as shown recently in the case of another TGF-β ligand [38].

Similarly, these findings on Panda could be correlated to the role of p38 in promoting *nodal* expression during D/V axis formation. As shown by Bradham and colleagues, p38 activity is required for *nodal* expression, and after a period of ubiquitous activation, it is specifically down-regulated on the presumptive dorsal side [29]. Panda, acting through Alk1/2 and Alk3/6, may be responsible for this down-regulation of p38 activity on the dorsal side, which may in turn prevent Nodal autoregulation, a hypothesis that we are currently testing.

### Maternal Determinants of D/V Axis Formation and Developmental Plasticity of the Early Blastomeres

The sea urchin embryo is well known for its remarkable developmental plasticity, the best example of this flexibility being the ability of each blastomere of the four-cell stage to regulate and to develop into smaller but normally patterned pluteus larvae. The outcome of this experiment deeply influenced ideas about how the D/V axis may be specified in this embryo, leading to the commonly accepted view that D/V patterning of the sea urchin embryo relies on cell interactions in the zygote and not on asymmetrically distributed determinants. On the other hand, classical experiments of Horstadius using unfertilized eggs showed that artificially activated meridional halves frequently differentiate as left-right or D/V pairs. These observations led Horstadius to conclude that "there seems to be no doubt as to the existence of a ventral-dorsal axis in the unfertilized sea urchin egg" [14]. The finding that the spatially restricted activity of Panda directs D/V axis formation strongly supports this conclusion. However, the finding that the spatially restricted activity of maternal *panda* mRNA directs the orientation of the D/V axis may seem at odds with the results of the Driesch experiment. How can we reconcile the fact that the first blastomeres show an equivalent potential to reestablish a secondary axis with the graded activity of a maternal factor controlling formation of the D/V axis in the early embryo? Following dissociation, each blastomere is expected to inherit a portion of the gradient of activity of Panda. One possibility is therefore that the portion of the gradient of activity of Panda inherited by each blastomere following dissociation is sufficient to reestablish the secondary axis. Indeed, with a reduced gradient of activity of Panda, the reaction-diffusion mechanism between Nodal and Lefty may in some cases be sufficient to amplify an initial asymmetry of the expression of *nodal* or *lefty*, leading to the restriction of *nodal* expression and to reestablishment of the secondary axis.

Hörstadius repeated and extended the Driesch experiment by rearing each of the four blastomeres in a separate dish [39]. Interestingly, he noted that in some cases one or two embryos of the quartet differentiated and established a D/V axis faster than the others. This is exactly what would be expected if the blastomeres inherit different portions of the gradient of activity of Panda. The results of Hörstadius are therefore consistent with our finding that there is a maternal gradient of a dorsalizing activity in the early embryo.

### An Antagonism between Nodal Signaling and Signaling from the BMP Type I Receptors as an Ancestral Mechanism Used to Specify the D/V Axis?

Our finding that the activity of two type I BMP receptors is essential to restrict *nodal* expression in the sea urchin embryo adds further support to the previously suggested idea that an antagonism between Nodal and BMP signaling may be an ancestral mechanism to specify the axes [40]. Evidence is accumulating that both in chordates and in echinoderms, a correct balance between BMP signals and Nodal signals is required for normal D/V patterning [10,11,40–42]. Both in echinoderms and in vertebrates, inactivation of the BMP pathway promotes cell fates controlled by Nodal. The process of specification of the distal visceral endoderm in the mouse embryo offers a striking example of such an antagonism. Formation and positioning of the distal visceral endoderm is regulated by an antagonism between BMP-Smad1 and Activin/Nodal-Smad2 signaling, and activin receptor II (ACVRII) has been shown to act as a limiting factor in this process [43,44]. Similarly, there is accumulating evidence that during left-right axis specification, the opposing activities of Nodal and BMPs are required for proper patterning along this axis and that BMP signaling is required to spatially restrict *nodal* expression [45–47]. For example, mouse embryos mutant for *smad1*, *smad5*, *spc4*, or for the gene encoding the BMP type I receptor ACVR1 display bilateral expression of *nodal* [48–51]. Intriguingly, although this antagonism may be fundamental for axis specification, the underlying mechanism is not well understood, and how the two pathways interact is not known. This antagonism may result from a direct interaction at the level of the signaling components. For example, it has been suggested that a competition at the level of Smad4 may set a threshold on Nodal signaling [52]. Alternatively, this antagonism may result from an interplay at the level of the ligands and secreted antagonists produced downstream of each pathway or at the level of ACVRII, which acts as a common receptor for both pathways. Finally, an antagonism at the level of the transcription factors induced by Nodal or BMP may be responsible for the antagonism between the two signaling pathways. Interestingly, a recent study proposed that the gene encoding the homeobox repressor Hbox12, a member of the Hbox12/pmar1/micro1 family [53–59], is expressed early on the dorsal side of the embryo and that it represses *nodal* expression [60], raising the possibility that this gene may act downstream of Panda to repress *nodal* expression. However, preliminary experiments to test this idea did not provide evidence for a link between Panda and Hbox12 (S8 Fig).

### Mechanism of Panda Inhibition of Nodal Signaling

Although we have detected expanded phospho-Smad signaling following misexpression of *panda* at the beginning of gastrulation, we failed to detect activation of Smad1/5/8 signaling during the early cleavage period, i.e., when *panda* is supposed to work, in embryos overexpressing *panda*. Therefore, Panda may not activate pSmad1/5/8 signaling directly, and the mechanism by which Panda antagonizes Nodal signaling during early stages remains presently unclear. We can envision several scenarios. Panda may antagonize Nodal signaling by heterodimerizing with Nodal and blocking its function. Such an activity has been reported in the case of BMP7 as well as in the case of Lefty [61,62]. Another possibility for the mechanism by which Panda may antagonize Nodal is that Panda may work like the Nodal antagonist Lefty, by sequestering a factor essential for Nodal signaling such as the co-receptor Cripto, ACVRII, or Alk4/5/7 [63]. The finding that blocking locally Nodal or ACVRII fully mimics the effects of overexpressing *panda* on the orientation of the D/V axis is consistent with this idea. However, these two hypotheses both predict that overexpression of Panda should strongly antagonize Nodal signaling, an activity that is not observed following overexpression of *panda*. One possibility to explain this result is that Panda may require another factor to act as a strong antagonist of Nodal signaling when overexpressed. Panda may therefore antagonize Nodal signaling in a way similar to that of Inhibin, which disrupts Activin signaling by acting through an intermediary factor and sequesters a factor required for Activin signaling [64]. A major difference between Panda and Inhibin is that while Inhibins have never been shown to require any type I receptor to function, our results indicate that Panda most likely requires the two BMP type I receptors Alk1/2 and Alk3/6 to antagonize Nodal. Therefore, if Panda acts by sequestering a factor required for Nodal signaling, this activity may also require functional Alk1/2 and Alk3/6, possibly in a complex with these two receptors. Finally, it remains also possible that Panda signals through these type I receptors and activates a noncanonical non-Smad pathway [65] that in turn may antagonize the Nodal pathway. In line with this conclusion, members of the Panda/Maverick/GDF15 subfamily lack a highly conserved leucine residue present in the so-called "wrist" domain of all BMP ligands (Leu 51 in human BMP2) that is critically required for binding of these factors to the type I BMP receptor. This suggests that members of the Panda/Maverick/GDF15 subfamily are low-affinity ligands for the BMP type I receptors or that the interaction between members of this subfamily and the BMP type I receptor may involve residues different from those involved in the interaction between canonical BMP ligands and the BMP type I receptors [66]. Also along these lines, it is intriguing to note that the mechanisms by which vertebrate GDF15 and *Drosophila* Maverick work remain also largely unknown. During *Drosophila* development, the *maverick* gene is broadly expressed during oogenesis and embryogenesis and throughout the larval stages [67]. Its function has long been enigmatic, but recent studies have uncovered a key role for Maverick during synaptogenesis at the neuromuscular junctions [68]. Maverick produced by glial cells was shown to promote expression of Glass bottom boat (Gbb), the fly ortholog of BMP7, in muscles. Similarly, the function of GDF15 in mice and humans is poorly understood. GDF15 is weakly expressed in most tissues, but its expression is induced in response to tissue injury, notably in the heart following myocardial infarction [69]. Neither *Drosophila* Maverick nor vertebrate GDF15 have been shown so far to activate any of the signaling pathways normally activated by BMP or Activin type ligands, and the mechanism by which these factors work remains unknown [69–71]. Our results showing that Panda antagonizes *nodal* expression by acting through type I BMP receptors and that overexpressed Panda induces *tbx2/3* without detectably activating Smad1/5/8 signaling points to non-Smad signaling as a potential mechanism for the crosstalk between the Nodal and BMP pathway [37]. However, we cannot completely rule out the possibility that Panda may induce a level of Smad1/5/8 activation below the current limits of detection, a level that would be sufficient to mediate its effects. Finally, a combination of the different mechanisms mentioned above including antagonism between Smad1 and Smad2, sequestration of rate-limiting components, and antagonism between transcription factors induced downstream of Smads may underlie the antagonism between Nodal and BMP signaling in the sea urchin embryo. Biochemical and functional experiments will therefore be required to dissect the mechanism by which Panda antagonizes Nodal signaling in the sea urchin embryo.

### Distinct and Sequential Roles for Panda, Lefty, and BMP2/4 during D/V Axis Formation

An interesting parallel can be drawn between the identification of Panda as a maternal TGF-β ligand acting through BMP receptors that cooperates with the zygotic BMP2/4 and the finding that maternal Univin, a Vg1 related ligand, cooperates with the zygotic Nodal. In the case of Nodal and Univin, it has been suggested that Nodal may heterodimerize with Univin and increase its specific activity [72]. Indeed, while Nodal is a strong ventralizing factor, overexpression of Univin has very modest effects on D/V patterning. Similarly, BMP2/4 has an extremely strong dorsalizing activity, while Panda essentially lacks dorsalizing activity. Heterodimer formation is, however, probably not the mechanism by which Panda and BMP2/4 cooperate, since Panda and BMP2/4 act at different periods during D/V axis formation and the activities of these factors appear to be qualitatively different. Panda is required early, starting at cleavage stages, well before BMP2/4 starts to be expressed, for the spatial restriction of *nodal* expression, while BMP2/4 is required much later, starting after hatching. Furthermore, while the only known activity of Panda is to limit and orient *nodal* expression and to induce *tbx2/3*, BMP2/4 has a key role in activating a cohort of dorsally expressed transcription factors and signaling molecules. Finally, while BMP2/4 strongly activates phosphorylation and nuclear translocation of Smad1/5/8, Panda only appears capable of weakly activating pSmad signaling. Therefore, D/V axis specification in the sea urchin embryo requires two phases of signaling from the BMP receptors, but these two phases are temporally and qualitatively different. The first phase of signaling, which covers the period of cleavage up to hatching blastula, is the consequence of maternal Panda signaling through Alk3/6 and Alk1/2, either through very low canonical Smad signaling or possibly through noncanonical Smad signaling, while the second phase, which starts after hatching and continues late in gastrulation, is the result of zygotically produced BMP2/4 factors binding to the same receptors but activating canonical phospho-Smad signaling.

Despite the fact that Panda and Lefty are both expressed early and that both factors are required nonredundantly to restrict *nodal* expression, the function of Panda is also clearly different from that of Lefty. Panda is capable of orienting the D/V axis when expressed into one blastomere at the two-cell stage, but overexpression of Panda in the egg does not suppress Nodal signaling. Furthermore, Panda is not sufficient to restrict *nodal* expression in *lefty* morphants. Therefore, the function of Panda appears to be to break the radial symmetry and to create the asymmetry of *nodal* expression rather than to maintain the asymmetry of *nodal* expression. In support of this idea, in the absence of Panda, *nodal* remains radially expressed up to the beginning of gastrulation. Therefore, although the function of Lefty is normal in these embryos, its activity is not sufficient to restrict *nodal* expression in the absence of Panda. In other words, without Panda, Lefty is unable to create an asymmetry of *nodal* expression. The function of Lefty appears therefore important to maintain the asymmetry of *nodal* expression previously established by Panda rather than to create this asymmetry.

### A Revised Model of D/V Axis Specification: Panda Breaks the Radial Symmetry by Restricting Nodal Expression

**Fig 11:**
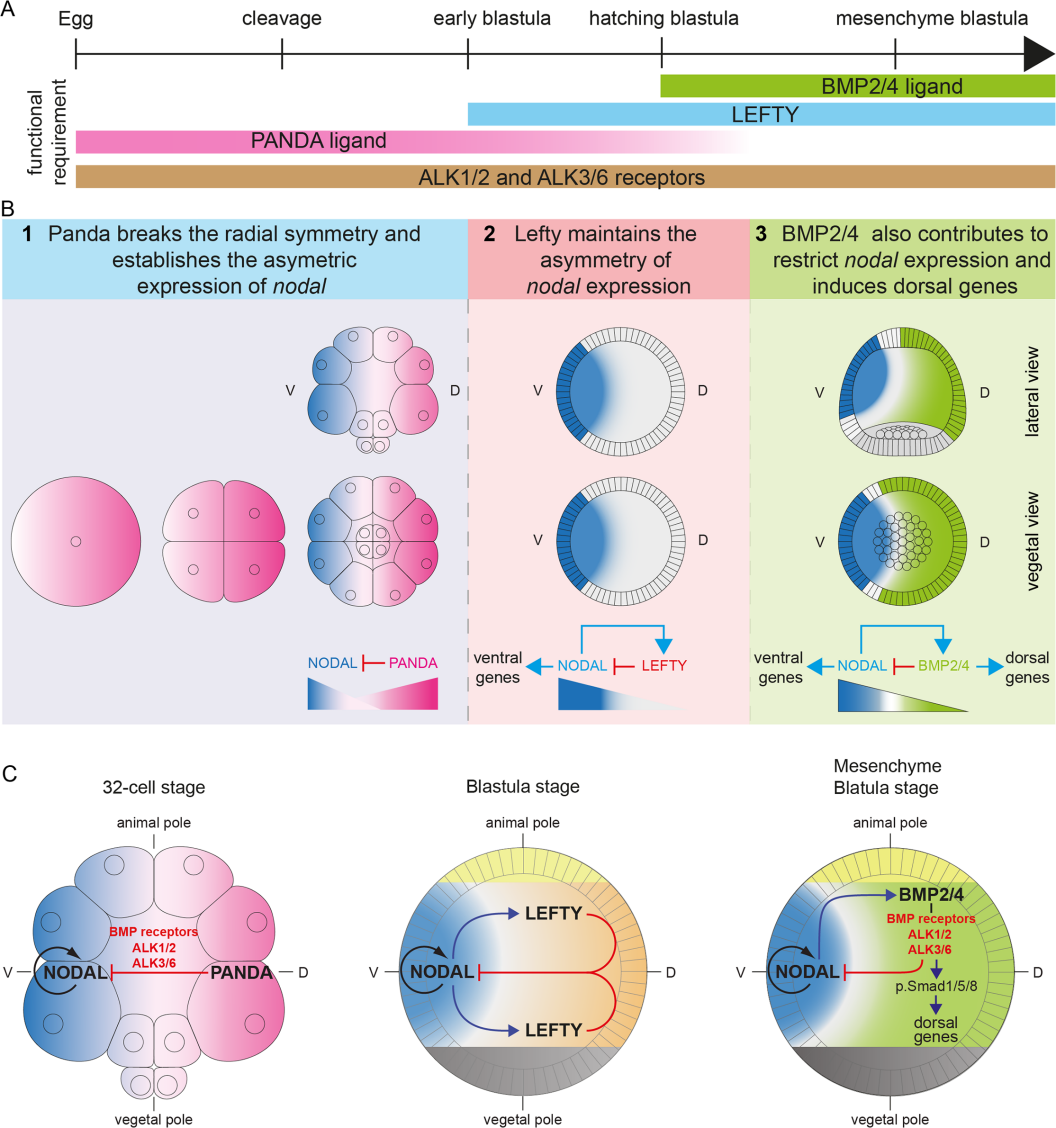
Model of D/V axis formation: The sequential activities of Panda, Lefty, and BMP2/4 establish the D/V axis. (A) Expression and functional requirements of the main ligands and receptors involved in D/V patterning. (B,C) The sequential activities of Panda, Lefty, and BMP2/4 progressively define the D/V axis. (1) During cleavage, a gradient of maternal Panda activity, acting through Alk3/6 and Alk1/2, first antagonizes *nodal* expression and breaks the radial symmetry of the embryo. (2) At the early blastula stage (7 h 30 hpf), following this initial symmetry breaking, spatially restricted Nodal induces Lefty, and Lefty, which diffuses more than Nodal, prevents Nodal autoregulation outside the presumptive ventral ectoderm. This second phase of Nodal antagonism is required to maintain the spatial restriction of *nodal* expression. (3) Starting at the hatching blastula stage (9 hpf), Nodal induces BMP2/4, and BMP2/4 signaling synergizes with Lefty to antagonize Nodal on the dorsal side. V, ventral; D, dorsal.

We have identified the maternal TGF-β ligand Panda as a novel and central player of the pathway controlling D/V axis formation. Although previous models placed Nodal as the first extracellular signal conveying spatial information for D/V axis formation, we can now place maternal Panda as the earliest known signal orienting the D/V axis upstream of *nodal* expression. A new model of D/V axis formation in the sea urchin embryo is the following (Fig 11). During oogenesis, maternal *panda* mRNA is deposited into the egg, possibly in a graded manner along the D/V axis, and following fertilization, this gradient of mRNA is translated into a shallow gradient of Panda protein. Starting at the 32/60-cell stage, ubiquitously expressed maternal transcription factors and maternal Wnt and Univin signaling activate *nodal* expression very broadly in the presumptive ectoderm. However, on the presumptive dorsal side, the increased activity of Panda weakly antagonizes *nodal* expression, introducing a first bias in *nodal* autoregulation that will initiate the spatial restriction of *nodal* expression to the presumptive ventral side. Then, starting at the early blastula stage, Nodal signaling induces expression of *lefty*, and the reaction-diffusion mechanism between Nodal and Lefty further contributes to maintain the spatial restriction of *nodal* expression. Finally, at the prehatching blastula stage, Nodal induces *bmp2/4* and *chordin* expression. Chordin prevents BMP2/4 signaling on the ventral side while it shuttles BMP2/4 to the opposite dorsal side, where BMP2/4 activates the gene regulatory network responsible for specification of the dorsal side of the embryo.

In conclusion, although Nodal remains the pivotal factor that regulates D/V axis formation in the sea urchin embryo, we have shown that the reaction-diffusion mechanism between Nodal and Lefty is not sufficient to break the radial symmetry of the embryo. This process of symmetry breaking is accomplished by a maternal factor, Panda, whose activity is required early and locally in the embryo to restrict the spatial expression of *nodal*. This work therefore illustrates how in the highly regulative sea urchin embryo, the secondary axis is already "penciled in " by the graded maternal information deposited into the egg in the form of a gradient of *panda* mRNA. Since *nodal* plays a key role in specification of the proximal distal axis in mammals and in specification of the secondary and left-right axes in a number of species, this raises the question as to whether members of the Panda/Maverick/GDF15 also provide a blueprint of axial development in these embryos.

## Materials and Methods

### Animals, Embryos, and Treatments

Adult sea urchins (*Paracentrotus lividus*) were collected in the bay of Villefranche. Embryos were cultured as described in Lepage and Gache (1989, 1990) [73,74].

For immunostaining and in situ hybridization at early stages, fertilization envelopes were removed by adding 2 mM 3-amino-1,2,4 triazole 1 min before insemination to prevent hardening of this envelope, followed by filtration through a 75 μm nylon net. Treatments with recombinant BMP2/4 or Nodal proteins were performed by adding the recombinant protein diluted from stocks in 1 mM HCl, in 24-well plates containing about 1,000 embryos in 2 ml of artificial sea water [25]. Treatments with NiCl_2_ were performed by exposing embryos to 0.2–0.3 mM of chemical. All treatments were carried out from 30 min to 48 h post fertilization.

### Cloning of the *panda* and *alk1/2* cDNAs

A full-length *panda* cDNA was obtained by screening a cDNA library with conventional methods and sequencing the corresponding clones. A full-length *alk1/2* cDNA was identified from a collection of *P*. *lividus* expressed sequence tags (ESTs) http://octopus.obs-vlfr.fr/). The complete sequence of this clone was determined.

The accessions numbers of *panda* and *alk1/2* mRNA are KF498642 and KF498643.

To make pCS2 Alk1/2-Q225D, the CAG codon encoding Glutamine in position 225 of Alk1/2 was mutated to GAC by oligonucleotide-directed in vitro mutagenesis using the two following oligonucleotides:

Alk1/2-Q225D fw: 5ʹ-cgaacagtagcaagagacatcaaccttattcaac -3ʹ

Alk1/2-Q225D rev: 5ʹ- gttgaataaggttgatgtctcttgctactgttcg-3ʹ

### Phylogenetic Analysis

TGF-β sequences from deuterostomes (vertebrates, cephalochordates, hemichordates, tunicates, and echinoderms), from protostomes (arthropods and molluscs), and from cnidarians were recovered from Genebank (http://www.ncbi.nlm.nih.gov/) using well-characterized orthologs of each TGF-β family member from human or mouse. The list of accession numbers of the 162 sequences is provided in the Supplementary Materials (S1 Text). Full-length sequences were aligned using ClustalOmega with default parameters (http://www.ebi.ac.uk/Tools/msa/clustalo/), and gap optimization and obvious alignment error corrections were made using Bioedit 7.0.5.3 (http://www.mbio.ncsu.edu/BioEdit/bioedit.html). The full complement of TGF-β sequences was recovered and used in the analysis in the case of human, mouse, sea urchin, *Saccoglossus*, *Branchiostoma*, and *Drosophila*. However, only a subset of sequences from *Gallus*, *Xenopus*, *Danio*, *Ciona*, *Crassostrea*, *Platynereis*, *Hydra*, and *Nematostella* was included in the analysis. Trees were built either using the maximum likelihood method based on the Whelan and Goldman model [75] or with Mr. Bayes3.2, using the mixed model with two independent runs of 3 million generations [76,77]. In the case of the maximum likelihood analysis, the tree was calculated using PhyML [3] with substitution model WAG (http://atgc.lirmm.fr/phyml/). A consensus tree with a 45% cutoff value was derived from 500 bootstrap analysis using Mega 3.1 (http://www.megasoftware.net/). For the Bayesian analysis, consensus trees and posterior probabilities were calculated once the stationary phase was reached (the average standard deviation of split frequencies was below 0.01). Numbers above branches represent posterior probabilities, calculated from this consensus.

### In Situ Hybridization

The *nodal*, *chordin*, *foxA*, *foxG*, *tbx2/3*, *hox7*, and *onecut* probes have been described previously [24,25,34]. The *panda* probe was derived from a full-length cDNA cloned in Bluescript, while the *alk1/2* probe was derived from a full-length cDNA cloned in pSport-Sfi. Probes derived from pBluescript vectors were synthesized with T7 RNA polymerase after linearization of the plasmids by NotI, while probes derived from pSport were synthesized with SP6 polymerase after linearization with XmaI. Control and experimental embryos were developed for the same time in the same experiments. Double in situ hybridizations were performed following the procedure of Thisse [78]. Detection of the lineage tracer was performed using an antifluorescein antibody coupled to alkaline phosphatase and using Fastred as substrate.

### RT-PCR

For the time-course analysis of *panda* expression, total RNA from staged embryos was extracted by the method of Chomczynski and Sacchi [79] and treated with DNase I. cDNA synthesis and PCR were performed using standard procedures using 32–35 cycles of PCR [80]. For the characterization of the splice-blocking morpholino, RNA was extracted at the pluteus stage from batches of 400 embryos injected with increasing doses of the morpholino. Following treatment with DNase-I and phenol-chloroform extraction, RNA samples were reverse transcribed using the QuantiTect reverse transcription kit from Quiagen and following the instructions provided by the manufacturer. The in vivo specificity and efficiency of this morpholino were monitored via semiquantitative RT-PCR using 40 cycles of PCR. PCR primers flanking intron 1 were used to amplify the cDNA products generated in the presence of this splice-blocking oligonucleotide. Both the Phusion DNA polymerase and the kit long-expand PCR from Roche that allows amplification of long DNA fragments were used following the recommendations of the manufacturers. Primer pairs for the *panda* and *mkk3* transcripts were derived from the open reading frames (respectively 1,482 bp and 1,020 bp):

*panda-fwd*: 5ʹ-GGAAAATGGCTCGACGCACATTCC-3ʹ

*panda-rev*: 5ʹ-TGAGCAGCCGCAACTTTCTACGACCATATC-3ʹ

*mkk3-fwd*: 5ʹ-ATGGCGAGTAAAGGTAAAAAG-3ʹ

*mkk3-rev*: 5ʹ-TTAACTATTCTCCGGATCTCC-3ʹ

### Anti-phospho-Smad1/5/8 Immunostaining

The antibody we used is an anti-phospho-Smad1/5/8 from Cell Signaling (Ref 9511) raised against a synthetic phosphopeptide corresponding to residues surrounding Ser463/465 contained in the motif SSVS of human Smad5. Embryos were fixed in paraformaldehyde 4% in microfiltrated sea water (MFSW) for 15 min and then briefly permeabilized with methanol. Embryos were rinsed once with Phosphate Buffered Saline Tween (PBST), four times with PBST–bovine serum albumine (BSA) 2%, and incubated overnight at +4°C with the primary antibody diluted 1/400 in PBST supplemented with 2% BSA. Embryos were washed six times with PBST-BSA 2%, and then the secondary antibody diluted in PBST-BSA 2% was added to the embryos. In all cases, the antibody was incubated overnight at +4°C. For immunofluorescence, the secondary antibody was washed six times with PBST. Two last rinses were made with PBST-Glycerol 25% and 50%, respectively. Embryos were mounted in a drop of the Citifluor antibleaching mounting medium and then observed under a conventional fluorescence microscope or with a confocal microscope. For Alkaline phosphatase revelation, two rinses were made with PBST following the secondary antibody incubation, and two with Tris Buffered Saline Tween (TBST). Embryos were washed twice with the alkaline phosphatase buffer supplemented with Tween 0.1%, and staining was performed with nitro blue tetrazolium (NBT) and 5-bromo-4-chloro-3-indolyl phosphate (BCIP) as substrates at the final concentration of 50 mM each. In both cases, staining was stopped by four rinses with PBST + EDTA 5 mM and then two rinses with PBST containing glycerol at 25% and 50%. Embryos were mounted and observed with a DIC microscope.

### Western Blotting

Protein samples (20 μg/lane) were separated by SDS-gel electrophoresis and electrophoretically transferred to 0.2 μm PVDF filters. After blocking for 2 h with 5% milk in TBST, blots were incubated overnight with the anti-phospho-Smad1/5/8 antibody (Ref 9511) diluted 1/1,000 in BSA 5% in TBST. After washing and incubation with the secondary antibody, bound antibodies were revealed by ECL immunodetection using the SuperSignal West Pico Chemiluminescent substrate (Pierce).

### Overexpression Analysis and Morpholino Injections

For overexpression studies, the coding sequence of the genes analyzed was amplified by PCR with a high-fidelity DNA polymerase using oligonucleotides containing restriction sites and cloned into pCS2. Capped mRNAs were synthesized from NotI-linearized templates using mMessage mMachine kit (Ambion). After synthesis, capped RNAs were purified on Sephadex G50 columns and quantitated by spectrophotometry. RNAs were mixed with Rhodamine Lysine-Fixable Dextran (RLDX) (10,000 MW) or Fluoresceinated Lysine-Fixable Dextran (FLDX) (70,000 MW) at 5 mg/ml and injected in the concentration range of 100–2,000 μg/ml.

Wild-type *panda* and mutated *panda* mRNAs were injected at 1,000 μg/ml. mRNAs encoding the activated form of *alk3/6* and *alk1/2*, Alk3/6Q230D (Alk3/6QD), and Alk1/2Q225D (Alk1/2QD) [25] were injected at 200 μg/ml. *bmp2/4* and *nodal* mRNAs were injected at 400 μg/ml. To make the Panda and Alk1/2 rescue constructs, oligonucleotides containing nine mismatches in the sequences recognized by the morpholinos were used to amplify the coding sequences. The sequences of these oligonucleotides are as follows:

Panda-rescue: 5ʹ-CCCATCGATACC**ATG**GCGAGGCGTACGTTGCAGCGCTTGCAAGGGAGC-3ʹ

Alk1/2-rescue: 5ʹ-CCCGGATCCACC **ATG**GCCACCCGTCGTCTTGAGTTTATTTTTATACTTTTGG-3ʹ (mismatches underlined).

Morpholinos oligonucleotides were dissolved in sterile water and injected at the one-cell stage together with Tetramethyl Rhodamine Dextran (10,000 MW) or Fluorescein dextran (70,000 MW) at 5 mg/ml. For each morpholino, a dose-response curve was obtained, and a concentration at which the oligomer did not elicit nonspecific defect was chosen. Approximately 2–4 pl of oligonucleotide solution was used in most of the experiments described here. The sequences for morpholino oligonucleotides used in this study are as follows:

Panda-Mo-ATG: 5ʹ-ATCTTTGGAATGTGCGTCGAGCCAT-3ʹ

Panda-Mo1-splice: 5ʹ-TACTAATTTGGCGAGCCTACCTGTA-3'

Panda-Mo2-splice: 5'-CGGAGGTCCATCTGAACGAAAGAAA-3'

Panda-Mo-5' UTR: 5'-TTTCCTCGTGCTTGTAGAAATCTCC-3'

Alk3/6-Mo: 5'- TAGTGTTACATCTGTCGCCATATTC-3'

Alk1/2-Mo: 5'-TAAATTCTAGTCGTCGCGTCGCCAT-3'

BMP5/8-Mo: 5'-CTTGGAGAGAAAATAAGCATATTCC-3'

BMP2/4-Mo: 5'-GACCCCAGTTTGAGGTGGTAACCAT-3'

ADMP-Mo: 5'-ACACGAAAATAATCTCCATTGTCTT-3'

ACVRII-Mo-ATG: 5’- GGATCTTTCCCAGCCATTTCGGATA-3’

The *panda*, *alk3/6*, and *alk1/2* morpholinos were used at 1.2 mM, except the *panda* Mo1 splice, which was used at 2 mM. The *bmp2/4* and *bmp5/8* morpholinos were used at 0.3 mM. The *acvrII* and *admp* morpholinos were used at 1.5 and 0.8 mM, respectively.

All the injections were repeated multiple times, and for each experiment, >100 embryos were analyzed. Only representative phenotypes present in at least 80% of the injected embryos are presented.

## Supporting Information

S1 Data Sequence alignment of mature TGF-β sequences. This alignment represents a portion of the alignment of the full-length proteins corresponding to the mature ligand domain.
(TXT)

S1 Fig Spatial expression and functional analysis of Alk1/2. (A) Alk1/2 is expressed maternally and ubiquitously during cleavage and blastula stages. In situ hybridization with *alk1/2* probe at the indicated stages. (B) Rescue of the *alk1/2* morphant phenotype by coinjection of wild-type *alk1/2* mRNA. While embryos injected with the *alk1/2* morpholino alone develop with a partially radialized phenotype, embryos injected with both the *alk1/2* morpholino and a synthetic *alk1/2* mRNA containing nine mismatches in the sequence recognized by the morpholino (*alk1/2 mismatch*) develop into pluteus larvae, like control embryos and embryos injected with the *alk1/2 mismatch* mRNA. V, ventral; D, dorsal.
(TIF)

S2 Fig Antagonism between Nodal and BMP2/4 revealed by treatments with recombinant BMP4. (A) Morphology at 72 hpf of wild-type embryos and embryos treated with gradual doses of recombinant BMP4 protein. Embryos treated with BMP4 at 0.25 μg/ml and 0.35 μg/ml develop with a radial morphology, a straight archenteron and a thick ciliary band-like ectoderm in the animal half (black arrowhead). This phenotype is characteristic of a Nodal loss-of-function phenotype, caused, for example, following inhibition of Nodal or *ACVRII*. Treatments with BMP4 at 0.5 μg/ml cause full dorsalization. (B) Visualization of *nodal* expression by in situ hybridization in wild-type embryos and embryos treated with gradual doses of BMP4. Loss of *nodal* expression is observed in embryos treated with BMP4 from 0.25 μg/ml and at 0.5 μg/ml. V, ventral side; D, dorsal side; VV, vegetal pole view.
(TIF)

S3 Fig bmp2/4, bmp5/8 and admp do not work redundantly to restrict nodal expression during D/V axis formation in the sea urchin embryo. Visualization of *nodal* and *chordin* at mesenchyme blastula stage by in situ hybridization in wild-type embryos and in embryos injected with either the *bmp2/4*, *bmp5/8*, or *admp* morpholinos or with a combination of *bmp2/4* + *bmp5/8* morpholinos or with the triple combination of *bmp2/4* + *bmp5/8* + *admp* morpholinos. Simple, double, or triple inactivation of *bmp2/4*, *bmp5/8*, and *admp* has no visible effect on *nodal* and *chordin* expression, which remains restricted to the ventral side. V, ventral side; D, dorsal side; LV, lateral view; VV, vegetal pole view.
(TIF)

S4 Fig Tree generated with the maximum likelihood method. The tree corresponds to that presented in Fig 4 but without the bootstrap branch support and with branch lengths. It was built using the maximum likelihood method based on the Whelan and Goldman model [75] using PhyML [3] with substitution model WAG (http://atgc.lirmm.fr/phyml/). Numbers above branches represent approximate likelihood ratio values [3].
(PDF)

S5 Fig Structural analysis of Panda. Scheme describing the characteristic pattern of cysteines found in the mature forms of different subfamilies of TGF-β ligands. On the basis of their pattern of cysteines, TGF-β ligands can be classified in three major groups. The hallmark of members of the first group is a characteristic pattern of nine cysteines in the ligand domain. This group includes ligands such as Panda/Maverick, TGF-β, Myostatin, Inhibins, or GDF15. Members of the second family lack the sixth cysteine that is involved in dimer formation and therefore contain eight cysteines instead of nine. Members of this second family include ligands such as Lefty, GDF3, and GDF9/BMP15. All the other TGF-β ligands have a characteristic pattern of seven cysteines. This is the case for Nodal, for GDF1/univin, and for all members of the BMP family of TGF-β ligands.
(TIF)

S6 Fig Bayesian analysis of TGF-β sequences from deuterostomes. The tree was built using Mr. Bayes3.2 [76,77], using the mixed model with two independent runs of 3 million generations and using full-length TGF-β sequences from deuterostomes. Consensus trees and posterior probabilities were calculated once the stationary phase was reached (the average standard deviation of split frequencies was below 0.01). The Bayesian method confirms the grouping of *panda* within a specific subfamily of GDF15-related TGF-β.
(JPG)

S7 Fig RT-PCR analysis and in vivo analysis of the panda-splice blocking morpholinos. (A) Scheme of the *panda* locus. The position of the two splice-blocking morpholino oligonucleotides flanking the single intron of the *panda* primary transcript is indicated. (B) Injection of morpholino oligonucleotides targeting either the donor or acceptor splice junctions did not cause any D/V phenotype, and the embryos developed into normal pluteus larvae. The Panda Mo 1 splice-inhibiting morpholino oligonucleotide did not cause any toxicity when injected at relatively high doses (2 mM), while the Panda Mo 2 started to be toxic above 0.5 mM. (C) Molecular analysis of the Panda splice-blocking morpholino 1. Aliquots of the PCR reaction were run on a 1% agarose gel, and the gel was stained with Syber safe. Left panel: PCR using conditions for amplification of long fragments. Right panel: PCR using normal conditions. *mkk3* is used as a control for cDNA synthesis. The *panda* splice Mo1 was effective to reduce the level of the mature *panda* transcript to less than one-tenth its normal value (1/15 as estimated with the Quantity One software from Biorad). In conditions allowing amplification of long DNA fragments, a faint PCR product of about 8 kb was detected in cDNAs derived from embryos injected with the splice-blocking morpholino, possibly corresponding to the unspliced *panda* transcript retaining the 6.3 kb intron. However, in normal PCR conditions, although a drastic reduction of the mature *panda* transcript was observed, it was not accompanied by the appearance of any major splice variant.
(TIF)

S8 Fig The homeobox gene *hbox12*, a member of the *pmar1*/*micro1* family of transcriptional repressors, does not work downstream of Panda. (A) Sequence alignment between pMar1/Micro1/Hbox12 family members. The sequences of several Pmar1 family members from *Strongylocentrotus purpuratus*, as well as the sequence of Pmar1 from *Lytechinus variegatus*, and of Micro1 from *Hemicentrotus pulcherrimus* and Hbox12 from *P*. *lividus* were retrieved from NCBI and aligned using ClustalOmega. Partial or full-length sequences of five additional Hbox12 family members were deduced from a large contig of genomic sequence containing a cluster of nine *hbox12*-like genes from *Paracentrotus*. The table shows the percentage of identity between these proteins. Note that the sequence divergence between Hbox12 from *Paracentrotus* and pMar1 from *Strongylocentrotus* or Micro1 from *Hemicentrotus* is similar to that existing between pMar1 from *Lytechinus* and Pmar1 from *Strongylocentrotus* or Micro1 from *Hemicentrotus*. (B) Whole mount in situ hybridization with a probe derived from a pBluescript plasmid containing the original *hbox12* sequence [55] reveals expression of the gene predominantly in precursors of the primary mesenchyme lineage at the 32-cell stage and the early blastula stage. (C) Overexpression of *hbox12* triggers massive epithelial-mesenchymal transition, mimicking the phenotype caused by overexpression of *pmar1*. Embryos injected with *hbox12* mRNA at doses above or equal to 5 μg/ml develop normally up to the mesenchyme blastula stage when they start to burst from the vegetal pole region. (D) Embryos injected with *hbox12* mRNA at doses below 5 μg/ml develop normally. (E) *hbox12* mRNA at 2.5 μg/ml was injected, together with a lineage label, into one blastomere at the two-cell stage, and the position of clone was recorded at the prism stage. Local overerexpression of *hbox12* does not orient the D/V axis as would be predicted for overexpression of a regulator of *nodal* expression.
(TIF)

S9 Fig Distribution of *panda* mRNA in immature ovocytes analyzed by in situ hybridization. Immature oocytes were collected from gonads and analyzed for the expression of *panda* by in situ hybridization. Results are presented for a randomly selected set of 16 ovocytes. The position of the animal-vegetal axis of the ovocyte is revealed by the asymmetrical localization of the germinal vesicle. The germinal vesicle and its nucleolus are closer to the animal pole where the micropyle and polar bodies will form [39]. When possible, for each ovocyte, a lateral view showing the animal-vegetal axis as a dashed line (top image) and an axial view showing it as a dot (lower image) are shown. Note, however, that at this stage it is not possible to recognize the future D/V embryonic axis. The subcortical localization of the in situ hybridization signal suggests that *panda* mRNA could be localized to the egg cortex.
(TIF)

S1 Text List of taxa and accession numbers used in the phylogenetic analysis. (DOCX)

## Abbreviations

ACVRII: activin receptor II
ADMP: anti-dorsalizing morphogenetic protein
Alk: activin receptor-like kinase
BCIP: 5-bromo-4-chloro-3-indolyl phosphate
BMP: bone morphogenetic protein
BSA: bovine serum albumine
CNS: central nervous system
DIC: differential interference contrast
D/V: dorsal-ventral
EGF: epidermal growth factor
EST: expressed sequence tag
FLDX: Fluoresceinated Lysine-Fixable Dextran
Gbb: Glass bottom boat
GFP: green fluorescent protein
hpf: hours after fertilization
MAP kinase: mitogen activated protein kinase
MFSW: microfiltrated sea water
Mo: morpholino antisense oligonucleotide
NCBI: National Center for Biotechnology Information
Panda: *paracentrotus anti-nodal dorsal activity*
PBST: Phosphate Buffered Saline Tween
PCR: polymerase chain reaction
PMC: primary mesenchyme cells
RLDX: Rhodamine Lysine-Fixable Dextran
RT-PCR: reverse transcription polymerase chain reaction
TBST: Tris Buffered Saline Tween
TGF-β: transforming growth factor beta

## References

[1] Schupbach T. Germ line and soma cooperate during oogenesis to establish the dorsal-ventral pattern of egg shell and embryo in Drosophila melanogaster. Cell. 1987;49:699–707. 3107840 10.1016/0092-8674(87)90546-0

[2] Roth S, Stein D, Nusslein-Volhard C. A gradient of nuclear localization of the dorsal protein determines dorsoventral pattern in the Drosophila embryo. Cell. 1989 12 22;59(6):1189–202. 2688897 10.1016/0092-8674(89)90774-5

[3] Rushlow CA, Han K, Manley JL, Levine M. The graded distribution of the dorsal morphogen is initiated by selective nuclear transport in Drosophila. Cell. 1989 12 22;59(6):1165–77. 2598265 10.1016/0092-8674(89)90772-1

[4] Steward R. Relocalization of the dorsal protein from the cytoplasm to the nucleus correlates with its function. Cell. 1989 12 22;59(6):1179–88. 2598266 10.1016/0092-8674(89)90773-3

[5] Jesuthasan S, Stahle U. Dynamic microtubules and specification of the zebrafish embryonic axis. Curr Biol. 1997 1 1;7(1):31–42. 9024620 10.1016/s0960-9822(06)00025-x

[6] Mizuno T, Yamaha E, Wakahara M, Kuroiwa A, Takeda H. Mesoderm induction in zebrafish. Nature. 1996(383):131–2.8538762

[7] Ober EA, Schulte-Merker S. Signals from the yolk cell induce mesoderm, neuroectoderm, the trunk organizer, and the notochord in zebrafish. Dev Biol. 1999;215(2):167–81. 10545228 10.1006/dbio.1999.9455

[8] Tao Q, Yokota C, Puck H, Kofron M, Birsoy B, Yan D, et al Maternal wnt11 activates the canonical wnt signaling pathway required for axis formation in Xenopus embryos. Cell. 2005 3 25;120(6):857–71. 15797385 10.1016/j.cell.2005.01.013

[9] Weaver C, Kimelman D. Move it or lose it: axis specification in Xenopus. Development. 2004 8;131(15):3491–9. 15262887 10.1242/dev.01284

[10] Langdon YG, Mullins MC. Maternal and zygotic control of zebrafish dorsoventral axial patterning. Annu Rev Genet. 2011;45:357–77. 10.1146/annurev-genet-110410-132517 21942367

[11] Arnold SJ, Robertson EJ. Making a commitment: cell lineage allocation and axis patterning in the early mouse embryo. Nat Rev Mol Cell Biol. 2009 2;10(2):91–103. 10.1038/nrm2618 19129791

[12] Papaioannou VE, Mkandawire J, Biggers JD. Development and phenotypic variability of genetically identical half mouse embryos. Development. 1989 8;106(4):817–27. 2562672 10.1242/dev.106.4.817

[13] Frankenberg S, Zernicka-Goetz M. Breaking Radial Symmetry In: Stern C, editor. Gastrulation. New York: Cold Spring Harbor Laboratory Press; 2004.

[14] Horstadius S. Experimental Embryology of Echinoderms. Oxford: Clarendon Press; 1973.

[15] Driesch H. The potency of the first two cells in echinoderm development/ Oppenheimer BHWaJM, editor. New York: Hafner; 1892.

[16] Hörstadius S, Wolsky A. W Roux' Arch Ent Org. 1936;135:69. 10.1007/BF02569029 28354465

[17] Child CM. Formation and Reduction of Indophenol Blue in Development of an Echinoderm. Proc Natl Acad Sci U S A. 1941 11 15;27(11):523–8. 16588496 10.1073/pnas.27.11.523 PMC1078374

[18] Cinquin O. Fast-tracking morphogen diffusion. J Theor Biol. 2006 2 7;238(3):532–40. 16095625 10.1016/j.jtbi.2005.06.011

[19] Czihak G. Entwicklungsphysiologische Untersuchungen an Echininiden (Verteilung und bedeutung der Cytochomoxydase). Whilhem roux's Archiv EntwickMechOrg. 1963;154:272–92. 10.1007/BF00582031 28355025

[20] Pease DC. Echinoderm bilateral determination in chemical concentration gradients I. The effects of cyanide, fericyanide, iodoacetate, picrate,dinitrophenol,urethane,iodine, malonate, etc J Exp Zool. 1941;86:381–405.

[21] Coffman JA, McCarthy JJ, Dickey-Sims C, Robertson AJ. Oral-aboral axis specification in the sea urchin embryo II. Mitochondrial distribution and redox state contribute to establishing polarity in Strongylocentrotus purpuratus. Dev Biol. 2004 9 1;273(1):160–71. 15302605 10.1016/j.ydbio.2004.06.005

[22] Coffman JA, Denegre JM. Mitochondria, redox signaling and axis specification in metazoan embryos. Dev Biol. 2007 8 15;308(2):266–80. 17586486 10.1016/j.ydbio.2007.05.042

[23] Coffman JA, Coluccio A, Planchart A, Robertson AJ. Oral-aboral axis specification in the sea urchin embryo III. Role of mitochondrial redox signaling via H2O2. Dev Biol. 2009 6 1;330(1):123–30. 10.1016/j.ydbio.2009.03.017 19328778 PMC2748885

[24] Duboc V, Rottinger E, Besnardeau L, Lepage T. Nodal and BMP2/4 signaling organizes the oral-aboral axis of the sea urchin embryo. Dev Cell. 2004 3;6(3):397–410. 15030762 10.1016/s1534-5807(04)00056-5

[25] Lapraz F, Besnardeau L, Lepage T. Dorsal-ventral patterning in echinoderms: insights into the evolution of the BMP-Chordin signaling Network. PLoS Biol. 2009;7(11):1–25. 10.1371/journal.pbio.1000248 PMC2772021 19956794

[26] Range R, Lapraz F, Quirin M, Marro S, Besnardeau L, Lepage T. Cis-regulatory analysis of nodal and maternal control of dorsal-ventral axis formation by Univin, a TGF-{beta} related to Vg1. Development. 2007 10;134(20):3649–64. 17855430 10.1242/dev.007799

[27] Nam J, Su YH, Lee PY, Robertson AJ, Coffman JA, Davidson EH. Cis-regulatory control of the nodal gene, initiator of the sea urchin oral ectoderm gene network. Dev Biol. 2007 6 15;306(2):860–9. 17451671 10.1016/j.ydbio.2007.03.033 PMC2063469

[28] Duboc V, Lapraz F, Besnardeau L, Lepage T. Lefty acts as an essential modulator of Nodal activity during sea urchin oral-aboral axis formation. Dev Biol. 2008 8 1;320(1):49–59. 10.1016/j.ydbio.2008.04.012 18582858

[29] Bradham CA, McClay DR. p38 MAPK is essential for secondary axis specification and patterning in sea urchin embryos. Development. 2006 1;133(1):21–32. 16319119 10.1242/dev.02160

[30] Saudemont A, Haillot E, Mekpoh F, Bessodes N, Quirin M, Lapraz F, et al Ancestral regulatory circuits governing ectoderm patterning downstream of Nodal and BMP2/4 revealed by gene regulatory network analysis in an echinoderm. PLoS Genet. 2010;6(12):e1001259 10.1371/journal.pgen.1001259 21203442 PMC3009687

[31] Reversade B, De Robertis EM. Regulation of ADMP and BMP2/4/7 at opposite embryonic poles generates a self-regulating morphogenetic field. Cell. 2005 12 16;123(6):1147–60. 16360041 10.1016/j.cell.2005.08.047 PMC2292129

[32] Reversade B, Kuroda H, Lee H, Mays A, De Robertis EM. Depletion of Bmp2, Bmp4, Bmp7 and Spemann organizer signals induces massive brain formation in Xenopus embryos. Development. 2005 8;132(15):3381–92. 15975940 10.1242/dev.01901 PMC2278118

[33] Lapraz F, Rottinger E, Duboc V, Range R, Duloquin L, Walton K, et al RTK and TGF-beta signaling pathways genes in the sea urchin genome. Dev Biol. 2006 8 24;300:132–52. 17084834 10.1016/j.ydbio.2006.08.048 PMC12337106

[34] Duboc V, Lapraz F, Saudemont A, Bessodes N, Mekpoh F, Haillot E, et al Nodal and BMP2/4 pattern the mesoderm and endoderm during development of the sea urchin embryo. Development. 2010 1;137(2):223–35. 10.1242/dev.042531 20040489

[35] Chipman AD, Ferrier DE, Brena C, Qu J, Hughes DS, Schroder R, et al The first myriapod genome sequence reveals conservative arthropod gene content and genome organisation in the centipede Strigamia maritima. PLoS Biol. 2014 11;12(11):e1002005 10.1371/journal.pbio.1002005 25423365 PMC4244043

[36] Wei Z, Angerer RC, Angerer LM. A database of mRNA expression patterns for the sea urchin embryo. Dev Biol. 2006 12 1;300(1):476–84. 17007833 10.1016/j.ydbio.2006.08.034 PMC1762123

[37] Derynck R, Zhang YE. Smad-dependent and Smad-independent pathways in TGF-beta family signalling. Nature. 2003 10 9;425(6958):577–84. 14534577 10.1038/nature02006

[38] Wei Z, Salmon RM, Upton PD, Morrell NW, Li W. Regulation of Bone Morphogenetic Protein 9 (BMP9) by Redox-dependent Proteolysis. J Biol Chem. 2014 11 7;289(45):31150–9. 10.1074/jbc.M114.579771 25237187 PMC4223318

[39] Horstadius S. Experimental Embryology of Echinoderms. Oxford: Clarendon Press 1973.

[40] Onai T, Yu JK, Blitz IL, Cho KW, Holland LZ. Opposing Nodal/Vg1 and BMP signals mediate axial patterning in embryos of the basal chordate amphioxus. Dev Biol. 2010 5 19;344(1):377–89. 10.1016/j.ydbio.2010.05.016 20488174 PMC4781670

[41] Molina MD, de Croze N, Haillot E, Lepage T. Nodal: master and commander of the dorsal-ventral and left-right axes in the sea urchin embryo. Curr Opin Genet Dev. 2013 6 13; 23(4):445–453. 10.1016/j.gde.2013.04.010 23769944

[42] Niehrs C. On growth and form: a Cartesian coordinate system of Wnt and BMP signaling specifies bilaterian body axes. Development. 2010 3;137(6):845–57. 10.1242/dev.039651 20179091

[43] Yamamoto M, Beppu H, Takaoka K, Meno C, Li E, Miyazono K, et al Antagonism between Smad1 and Smad2 signaling determines the site of distal visceral endoderm formation in the mouse embryo. J Cell Biol. 2009 1 26;184(2):323–34. 10.1083/jcb.200808044 19153222 PMC2654303

[44] Schmierer B, Hill CS. TGFbeta-SMAD signal transduction: molecular specificity and functional flexibility. Nat Rev Mol Cell Biol. 2007 12;8(12):970–82. 18000526 10.1038/nrm2297

[45] Bessodes N, Haillot E, Duboc V, Rottinger E, Lahaye F, Lepage T. Reciprocal Signaling between the Ectoderm and a Mesendodermal Left-Right Organizer Directs Left-Right Determination in the Sea Urchin Embryo. PLoS Genet. 2012 12;8(12):e1003121 10.1371/journal.pgen.1003121 23271979 PMC3521660

[46] Luo YJ, Su YH. Opposing nodal and BMP signals regulate left-right asymmetry in the sea urchin larva. PLoS Biol. 2012 10;10(10):e1001402 10.1371/journal.pbio.1001402 23055827 PMC3467216

[47] Blum M, Feistel K, Thumberger T, Schweickert A. The evolution and conservation of left-right patterning mechanisms. Development. 2014 4;141(8):1603–13. 10.1242/dev.100560 24715452

[48] Chang H, Zwijsen A, Vogel H, Huylebroeck D, Matzuk MM. Smad5 is essential for left-right asymmetry in mice. Dev Biol. 2000;219(1):71–8. 10677256 10.1006/dbio.1999.9594

[49] Mine N, Anderson RM, Klingensmith J. BMP antagonism is required in both the node and lateral plate mesoderm for mammalian left-right axis establishment. Development. 2008 8;135(14):2425–34. 10.1242/dev.018986 18550712

[50] Constam DB, Robertson EJ. SPC4/PACE4 regulates a TGFbeta signaling network during axis formation. Genes Dev. 2000;14(9):1146–55. 10809672 PMC316583

[51] Kishigami S, Yoshikawa S, Castranio T, Okazaki K, Furuta Y, Mishina Y. BMP signaling through ACVRI is required for left-right patterning in the early mouse embryo. Dev Biol. 2004 12 1;276(1):185–93. 15531373 10.1016/j.ydbio.2004.08.042

[52] Furtado MB, Solloway MJ, Jones VJ, Costa MW, Biben C, Wolstein O, et al BMP/SMAD1 signaling sets a threshold for the left/right pathway in lateral plate mesoderm and limits availability of SMAD4. Genes Dev. 2008 11 1;22(21):3037–49. 10.1101/gad.1682108 18981480 PMC2577791

[53] Oliveri P, Carrick DM, Davidson EH. A regulatory gene network that directs micromere specification in the sea urchin embryo. Dev Biol. 2002;246(1):209–28. 12027443 10.1006/dbio.2002.0627

[54] Oliveri P, Davidson EH, McClay DR. Activation of pmar1 controls specification of micromeres in the sea urchin embryo. Dev Biol. 2003 6 1;258(1):32–43. 12781680 10.1016/s0012-1606(03)00108-8

[55] Di Bernardo M, Russo R, Oliveri P, Melfi R, Spinelli G. Homeobox-containing gene transiently expressed in a spatially restricted pattern in the early sea urchin embryo. Proc Natl Acad Sci U S A. 1995 8 29;92(18):8180–4. 7667265 10.1073/pnas.92.18.8180 PMC41120

[56] Kitamura K, Nishimura Y, Kubotera N, Higuchi Y, Yamaguchi M. Transient activation of the micro1 homeobox gene family in the sea urchin (Hemicentrotus pulcherrimus) micromere. Dev Genes Evol. 2002 2;212(1):1–10. 11875651 10.1007/s00427-001-0202-3

[57] Nishimura Y, Sato T, Morita Y, Yamazaki A, Akasaka K, Yamaguchi M. Structure, regulation, and function of micro1 in the sea urchin Hemicentrotus pulcherrimus. Dev Genes Evol. 2004 11;214(11):525–36. 15480758 10.1007/s00427-004-0442-0

[58] Yamazaki A, Kawabata R, Shiomi K, Amemiya S, Sawaguchi M, Mitsunaga-Nakatsubo K, et al The micro1 gene is necessary and sufficient for micromere differentiation and mid/hindgut-inducing activity in the sea urchin embryo. Dev Genes Evol. 2005 9;215(9):450–59. 16078091 10.1007/s00427-005-0006-y

[59] Yamazaki A, Ki S, Kokubo T, Yamaguchi M. Structure-function correlation of micro1 for micromere specification in sea urchin embryos. Mech Dev. 2009 Aug-Sep;126(8–9):611–23. 10.1016/j.mod.2009.06.1083 19549568

[60] Cavalieri V, Spinelli G. Early asymmetric cues triggering the dorsal/ventral gene regulatory network of the sea urchin embryo. Elife. 2014;3:e04664 10.7554/eLife.04664 25457050 PMC4273433

[61] Yeo C, Whitman M. Nodal signals to Smads through Cripto-dependent and Cripto-independent mechanisms. Mol Cell. 2001;7(5):949–57. 11389842 10.1016/s1097-2765(01)00249-0

[62] Chen C, Shen MM. Two modes by which Lefty proteins inhibit nodal signaling. Curr Biol. 2004 4 6;14(7):618–24. 15062104 10.1016/j.cub.2004.02.042

[63] Cheng SK, Olale F, Brivanlou AH, Schier AF. Lefty Blocks a Subset of TGFbeta Signals by Antagonizing EGF-CFC Coreceptors. PLoS Biol. 2004 2;2(2):E30 14966532 10.1371/journal.pbio.0020030 PMC340941

[64] Lewis KA, Gray PC, Blount AL, MacConell LA, Wiater E, Bilezikjian LM, et al Betaglycan binds inhibin and can mediate functional antagonism of activin signalling. Nature. 2000 3 23;404(6776):411–4. 10746731 10.1038/35006129

[65] Zhang YE. Non-Smad pathways in TGF-beta signaling. Cell Res. 2009 1;19(1):128–39. 10.1038/cr.2008.328 19114990 PMC2635127

[66] Keller S, Nickel J, Zhang JL, Sebald W, Mueller TD. Molecular recognition of BMP-2 and BMP receptor IA. Nat Struct Mol Biol. 2004 5;11(5):481–8. 15064755 10.1038/nsmb756

[67] Nguyen M, Parker L, Arora K. Identification of maverick, a novel member of the TGF-beta superfamily in Drosophila. Mech Dev. 2000 7;95(1–2):201–6. 10906462 10.1016/s0925-4773(00)00338-5

[68] Fuentes-Medel Y, Ashley J, Barria R, Maloney R, Freeman M, Budnik V. Integration of a retrograde signal during synapse formation by glia-secreted TGF-beta ligand. Curr Biol. 2012 10 9;22(19):1831–8. 10.1016/j.cub.2012.07.063 22959350 PMC3605899

[69] Kempf T, Zarbock A, Widera C, Butz S, Stadtmann A, Rossaint J, et al GDF-15 is an inhibitor of leukocyte integrin activation required for survival after myocardial infarction in mice. Nat Med. 2011 5;17(5):581–8. 10.1038/nm.2354 21516086

[70] Gesualdi SC, Haerry TE. Distinct signaling of Drosophila Activin/TGF-beta family members. Fly (Austin). 2007 Jul-Aug;1(4):212–21. 10.4161/fly.5116 18820452

[71] Hevia CF, de Celis JF. Activation and function of TGFbeta signalling during Drosophila wing development and its interactions with the BMP pathway. Dev Biol. 2013 5 1;377(1):138–53. 10.1016/j.ydbio.2013.02.004 23485686

[72] Tanaka C, Sakuma R, Nakamura T, Hamada H, Saijoh Y. Long-range action of Nodal requires interaction with GDF1. Genes Dev. 2007 12 15;21(24):3272–82. 18079174 10.1101/gad.1623907 PMC2113028

[73] Lepage T, Gache C. Purification and characterization of the sea urchin embryo hatching enzyme. J Biol Chem. 1989;264(9):4787–93. 2925668

[74] Lepage T, Gache C. Early expression of a collagenase-like hatching enzyme gene in the sea urchin embryo. Embo J. 1990;9(9):3003–12. 2167841 10.1002/j.1460-2075.1990.tb07493.x PMC552018

[75] Whelan S, Goldman N. A general empirical model of protein evolution derived from multiple protein families using a maximum-likelihood approach. Mol Biol Evol. 2001 5;18(5):691–9. 11319253 10.1093/oxfordjournals.molbev.a003851

[76] Ronquist F, Huelsenbeck JP. MrBayes 3: Bayesian phylogenetic inference under mixed models. Bioinformatics. 2003 8 12;19(12):1572–4. 12912839 10.1093/bioinformatics/btg180

[77] Ronquist F, Teslenko M, van der Mark P, Ayres DL, Darling A, Hohna S, et al MrBayes 3.2: efficient Bayesian phylogenetic inference and model choice across a large model space. Syst Biol. 2012 5;61(3):539–42. 10.1093/sysbio/sys029 22357727 PMC3329765

[78] Thisse B, Heyer V, Lux A, Alunni V, Degrave A, Seiliez I, et al Spatial and temporal expression of the zebrafish genome by large-scale in situ hybridization screening. Methods Cell Biol. 2004;77:505–19. 15602929 10.1016/s0091-679x(04)77027-2

[79] Chomczynski P, Sacchi N. Single-step method of RNA isolation by acid guanidinium thiocyanate- phenol-chloroform extraction. Anal Biochem. 1987;162(1):156–9. 2440339 10.1006/abio.1987.9999

[80] Sambrook J, Fritsch EF, Maniatis T. Molecular cloning A laboratory manual. 4th ed. Cold Spring Harbor, NY: Cold Spring Harbor Laboratory Press; 2012.

