# Supplementary figures and images for: The Maternal Maverick/GDF15-like TGF-β Ligand Panda Directs Dorsal-Ventral Axis Formation by Restricting Nodal Expression in the Sea Urchin Embryo
Source: PLoS Biol. 2015 Sep 9;13(9):e1002247. doi: 10.1371/journal.pbio.1002247 (PMC4564238; doi:10.1371/journal.pbio.1002247)

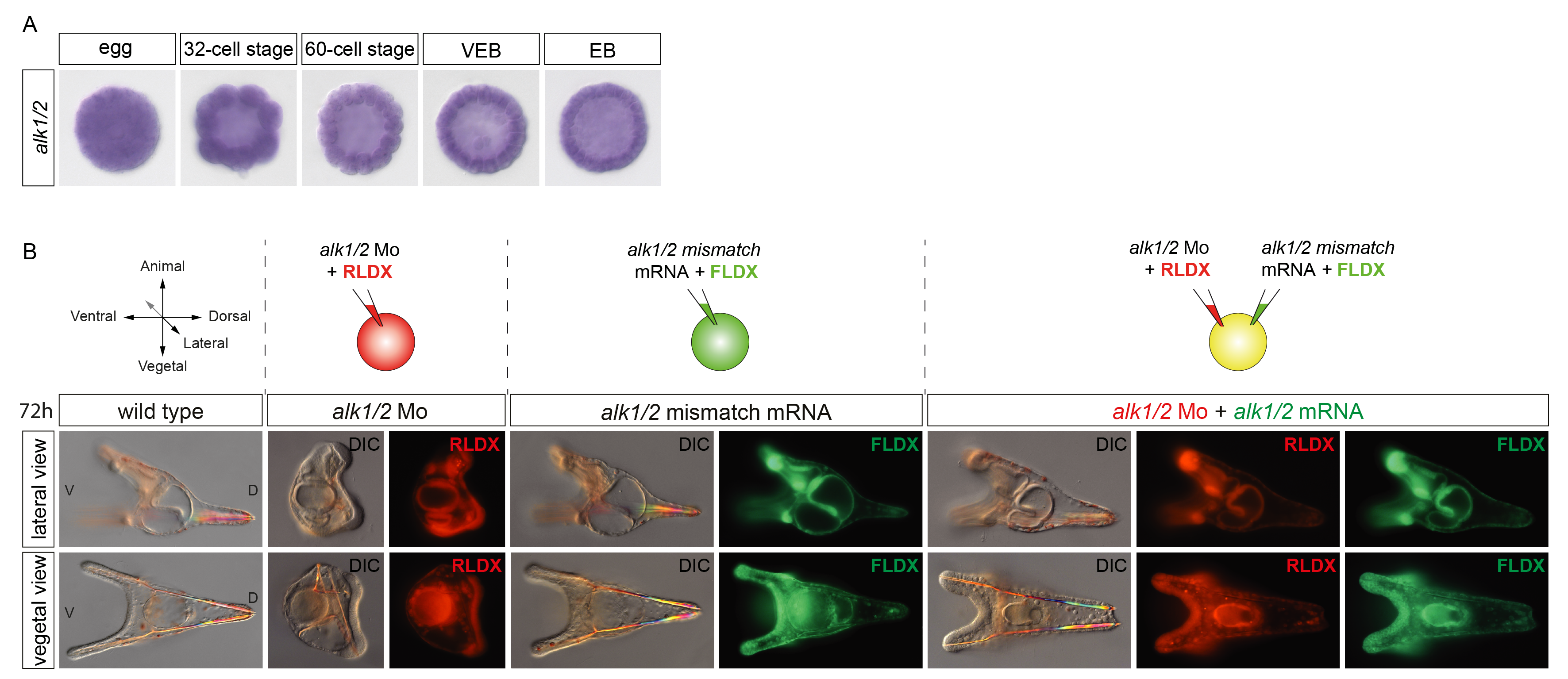

Supplement: S1 Fig — (A) Alk1/2 is expressed maternally and ubiquitously during cleavage and blastula stages. In situ hybridization with alk1/2 probe at the indicated stages. (B) Rescue of the alk1/2 morphant phenotype by coinjection of wild-type alk1/2 mRNA. While embryos injected with the alk1/2 morpholino alone develop with a partially radialized phenotype, embryos injected with both the alk1/2 morpholino and a synthetic alk1/2 mRNA containing nine mismatches in the sequence recognized by the morpholino (alk1/2 mismatch) develop into pluteus larvae, like control embryos and embryos injected with the alk1/2 mismatch mRNA. V, ventral; D, dorsal. (TIF) [file pbio.1002247.s002.tif]

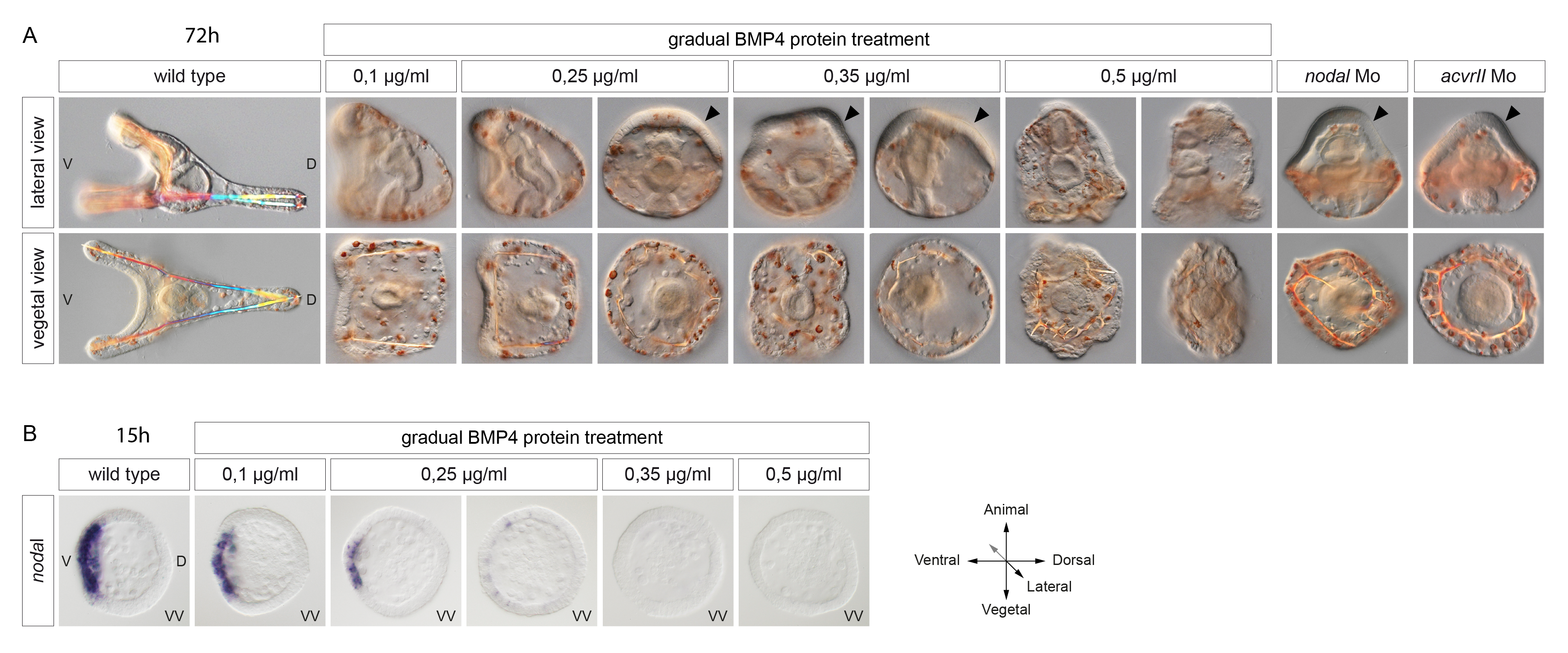

Supplement: S2 Fig — (A) Morphology at 72 hpf of wild-type embryos and embryos treated with gradual doses of recombinant BMP4 protein. Embryos treated with BMP4 at 0.25 μg/ml and 0.35 μg/ml develop with a radial morphology, a straight archenteron and a thick ciliary band-like ectoderm in the animal half (black arrowhead). This phenotype is characteristic of a Nodal loss-of-function phenotype, caused, for example, following inhibition of Nodal or ACVRII. Treatments with BMP4 at 0.5 μg/ml cause full dorsalization. (B) Visualization of nodal expression by in situ hybridization in wild-type embryos and embryos treated with gradual doses of BMP4. Loss of nodal expression is observed in embryos treated with BMP4 from 0.25 μg/ml and at 0.5 μg/ml. V, ventral side; D, dorsal side; VV, vegetal pole view. (TIF) [file pbio.1002247.s003.tif]

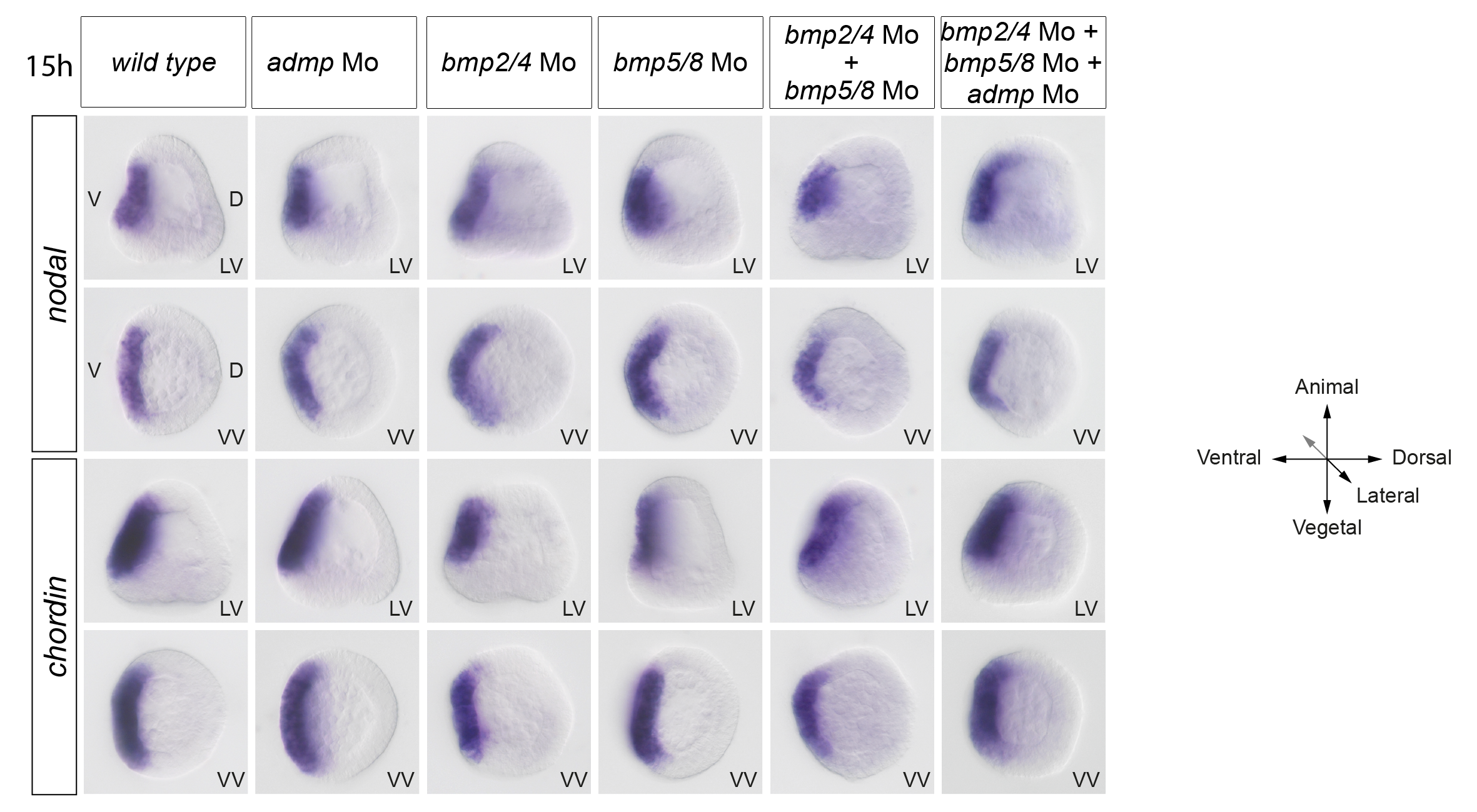

Supplement: S3 Fig — Visualization of nodal and chordin at mesenchyme blastula stage by in situ hybridization in wild-type embryos and in embryos injected with either the bmp2/4, bmp5/8, or admp morpholinos or with a combination of bmp2/4 + bmp5/8 morpholinos or with the triple combination of bmp2/4 + bmp5/8 + admp morpholinos. Simple, double, or triple inactivation of bmp2/4, bmp5/8, and admp has no visible effect on nodal and chordin expression, which remains restricted to the ventral side. V, ventral side; D, dorsal side; LV, lateral view; VV, vegetal pole view. (TIF) [file pbio.1002247.s004.tif]

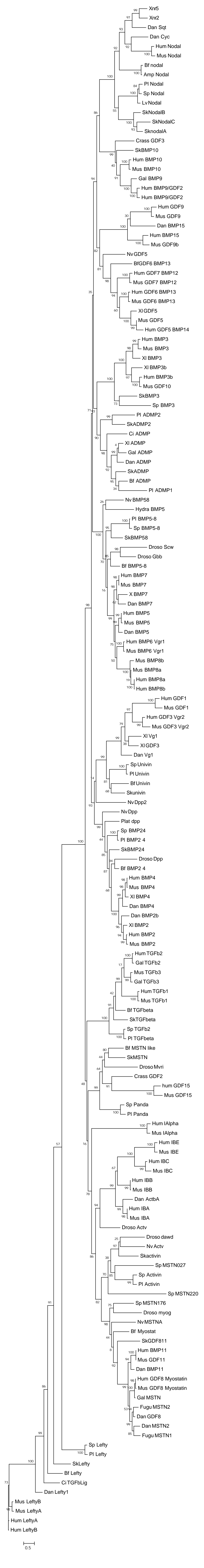

Supplement: S4 Fig — The tree corresponds to that presented in Fig 4 but without the bootstrap branch support and with branch lengths. It was built using the maximum likelihood method based on the Whelan and Goldman model [75] using PhyML [3] with substitution model WAG (http://atgc.lirmm.fr/phyml/). Numbers above branches represent approximate likelihood ratio values [3]. (PDF) [file pbio.1002247.s005.pdf]

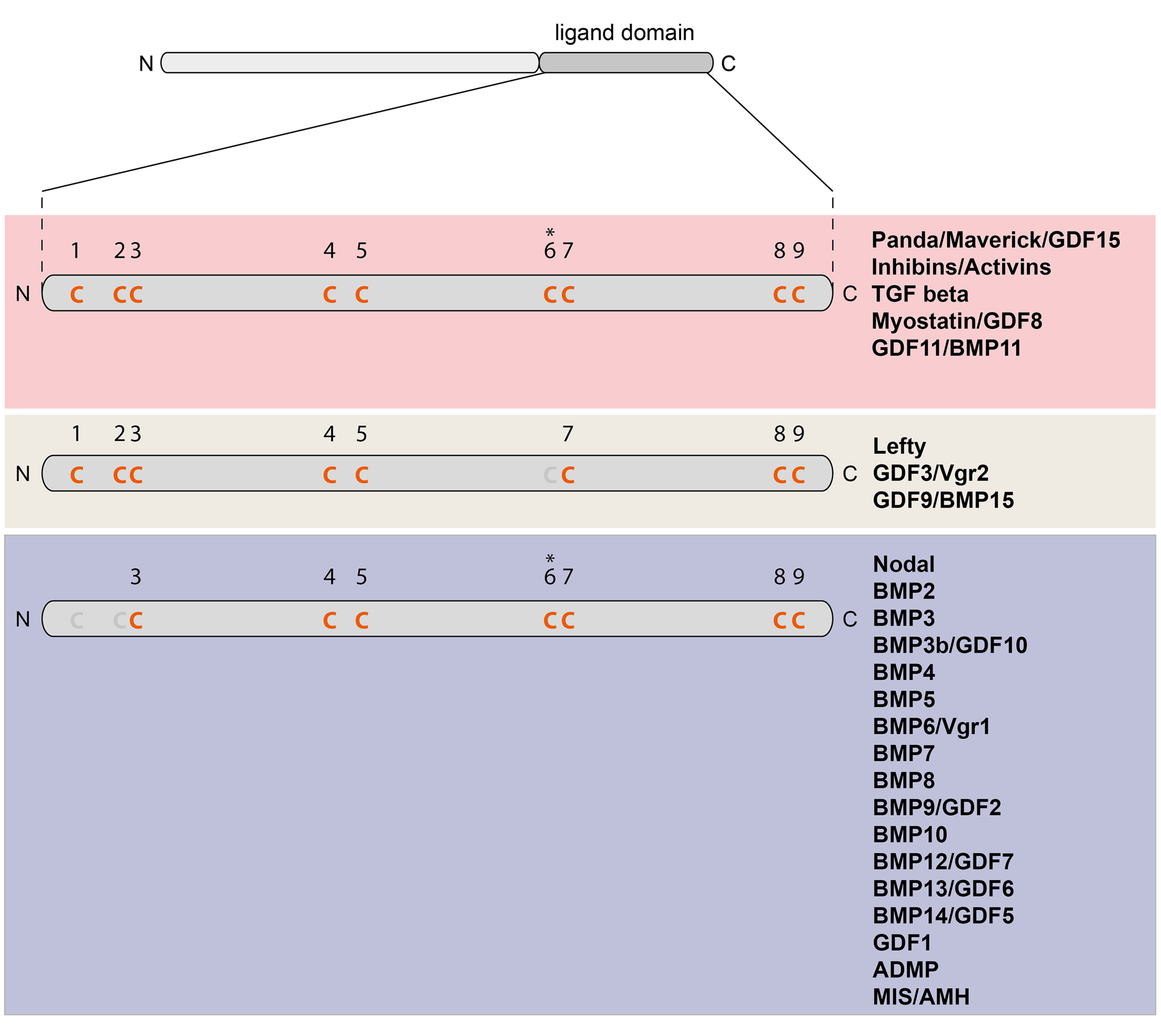

Supplement: S5 Fig — Scheme describing the characteristic pattern of cysteines found in the mature forms of different subfamilies of TGF-β ligands. On the basis of their pattern of cysteines, TGF-β ligands can be classified in three major groups. The hallmark of members of the first group is a characteristic pattern of nine cysteines in the ligand domain. This group includes ligands such as Panda/Maverick, TGF-β, Myostatin, Inhibins, or GDF15. Members of the second family lack the sixth cysteine that is involved in dimer formation and therefore contain eight cysteines instead of nine. Members of this second family include ligands such as Lefty, GDF3, and GDF9/BMP15. All the other TGF-β ligands have a characteristic pattern of seven cysteines. This is the case for Nodal, for GDF1/univin, and for all members of the BMP family of TGF-β ligands. (TIF) [file pbio.1002247.s006.tif]

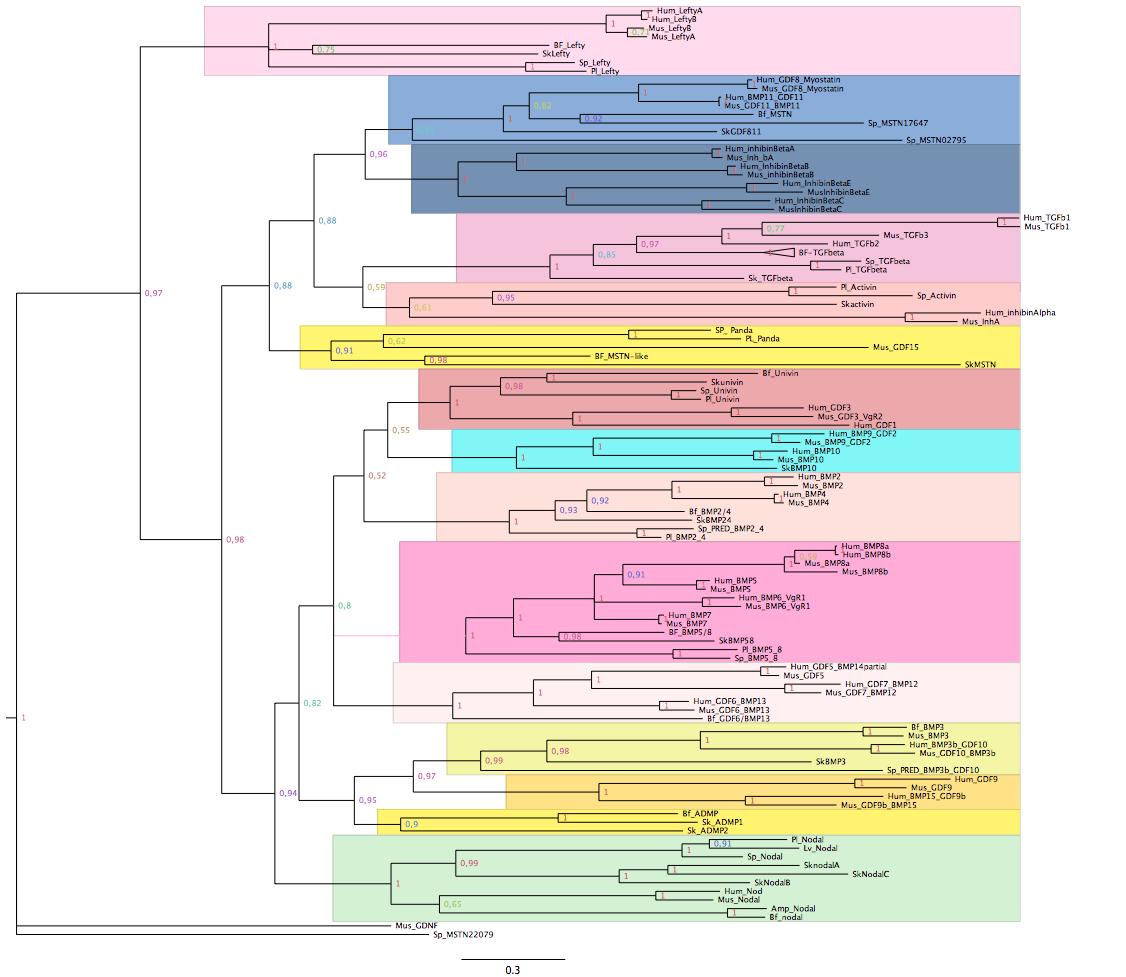

Supplement: S6 Fig — The tree was built using Mr. Bayes3.2 [76,77], using the mixed model with two independent runs of 3 million generations and using full-length TGF-β sequences from deuterostomes. Consensus trees and posterior probabilities were calculated once the stationary phase was reached (the average standard deviation of split frequencies was below 0.01). The Bayesian method confirms the grouping of panda within a specific subfamily of GDF15-related TGF-β. (JPG) [file pbio.1002247.s007.jpg]

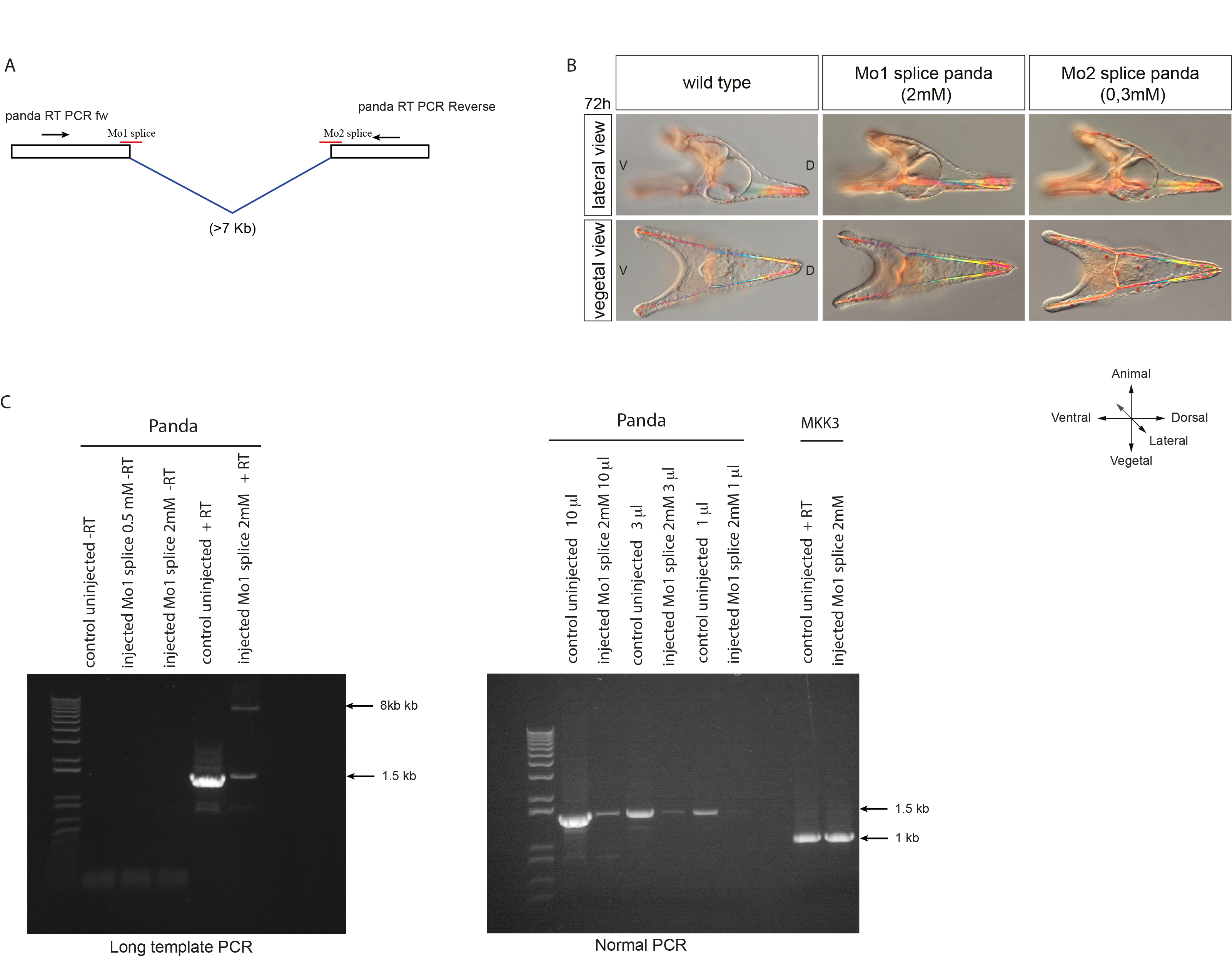

Supplement: S7 Fig — (A) Scheme of the panda locus. The position of the two splice-blocking morpholino oligonucleotides flanking the single intron of the panda primary transcript is indicated. (B) Injection of morpholino oligonucleotides targeting either the donor or acceptor splice junctions did not cause any D/V phenotype, and the embryos developed into normal pluteus larvae. The Panda Mo 1 splice-inhibiting morpholino oligonucleotide did not cause any toxicity when injected at relatively high doses (2 mM), while the Panda Mo 2 started to be toxic above 0.5 mM. (C) Molecular analysis of the Panda splice-blocking morpholino 1. Aliquots of the PCR reaction were run on a 1% agarose gel, and the gel was stained with Syber safe. Left panel: PCR using conditions for amplification of long fragments. Right panel: PCR using normal conditions. mkk3 is used as a control for cDNA synthesis. The panda splice Mo1 was effective to reduce the level of the mature panda transcript to less than one-tenth its normal value (1/15 as estimated with the Quantity One software from Biorad). In conditions allowing amplification of long DNA fragments, a faint PCR product of about 8 kb was detected in cDNAs derived from embryos injected with the splice-blocking morpholino, possibly corresponding to the unspliced panda transcript retaining the 6.3 kb intron. However, in normal PCR conditions, although a drastic reduction of the mature panda transcript was observed, it was not accompanied by the appearance of any major splice variant. (TIF) [file pbio.1002247.s008.tif]

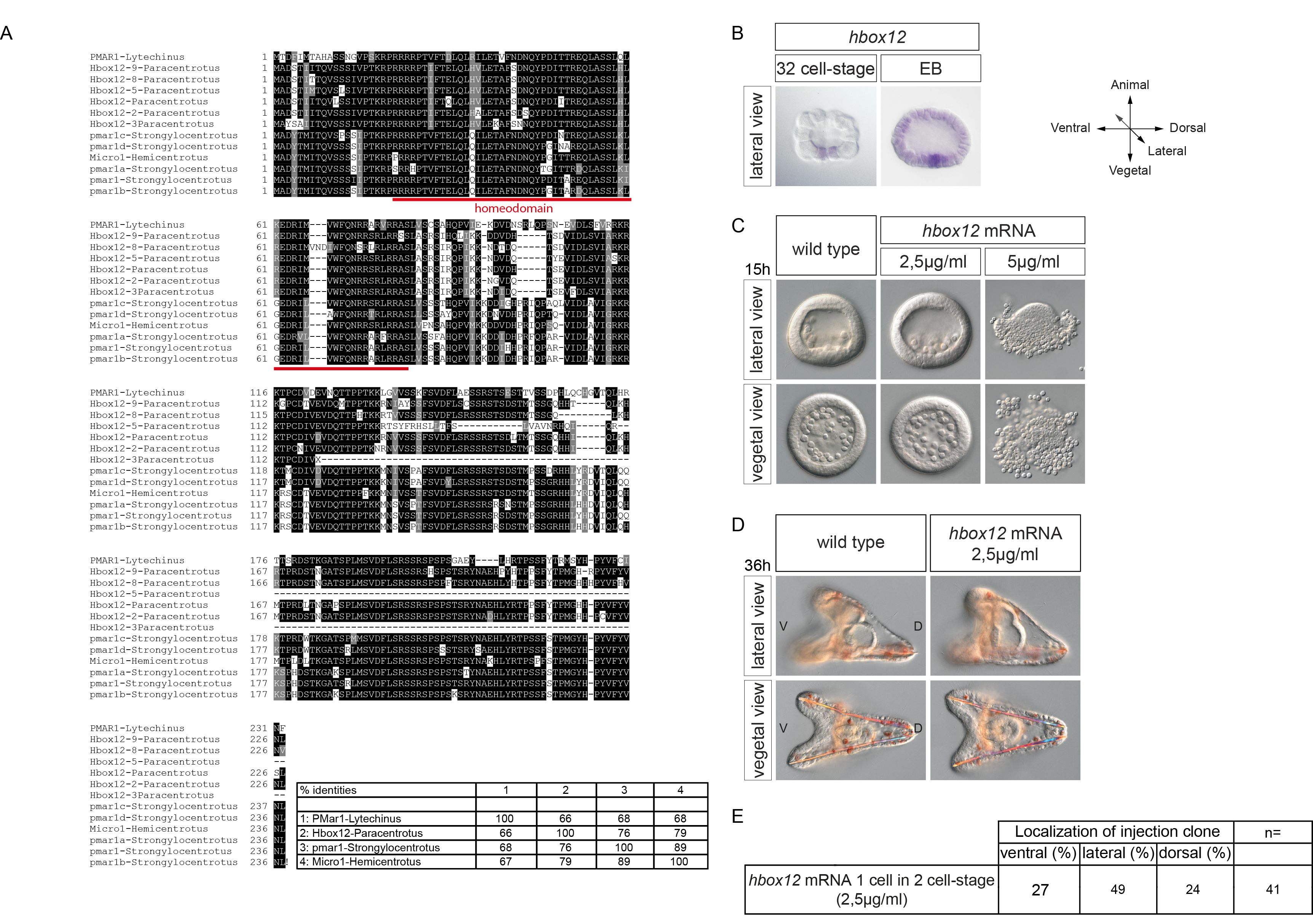

Supplement: S8 Fig — (A) Sequence alignment between pMar1/Micro1/Hbox12 family members. The sequences of several Pmar1 family members from Strongylocentrotus purpuratus, as well as the sequence of Pmar1 from Lytechinus variegatus, and of Micro1 from Hemicentrotus pulcherrimus and Hbox12 from P. lividus were retrieved from NCBI and aligned using ClustalOmega. Partial or full-length sequences of five additional Hbox12 family members were deduced from a large contig of genomic sequence containing a cluster of nine hbox12-like genes from Paracentrotus. The table shows the percentage of identity between these proteins. Note that the sequence divergence between Hbox12 from Paracentrotus and pMar1 from Strongylocentrotus or Micro1 from Hemicentrotus is similar to that existing between pMar1 from Lytechinus and Pmar1 from Strongylocentrotus or Micro1 from Hemicentrotus. (B) Whole mount in situ hybridization with a probe derived from a pBluescript plasmid containing the original hbox12 sequence [55] reveals expression of the gene predominantly in precursors of the primary mesenchyme lineage at the 32-cell stage and the early blastula stage. (C) Overexpression of hbox12 triggers massive epithelial-mesenchymal transition, mimicking the phenotype caused by overexpression of pmar1. Embryos injected with hbox12 mRNA at doses above or equal to 5 μg/ml develop normally up to the mesenchyme blastula stage when they start to burst from the vegetal pole region. (D) Embryos injected with hbox12 mRNA at doses below 5 μg/ml develop normally. (E) hbox12 mRNA at 2.5 μg/ml was injected, together with a lineage label, into one blastomere at the two-cell stage, and the position of clone was recorded at the prism stage. Local overerexpression of hbox12 does not orient the D/V axis as would be predicted for overexpression of a regulator of nodal expression. (TIF) [file pbio.1002247.s009.tif]

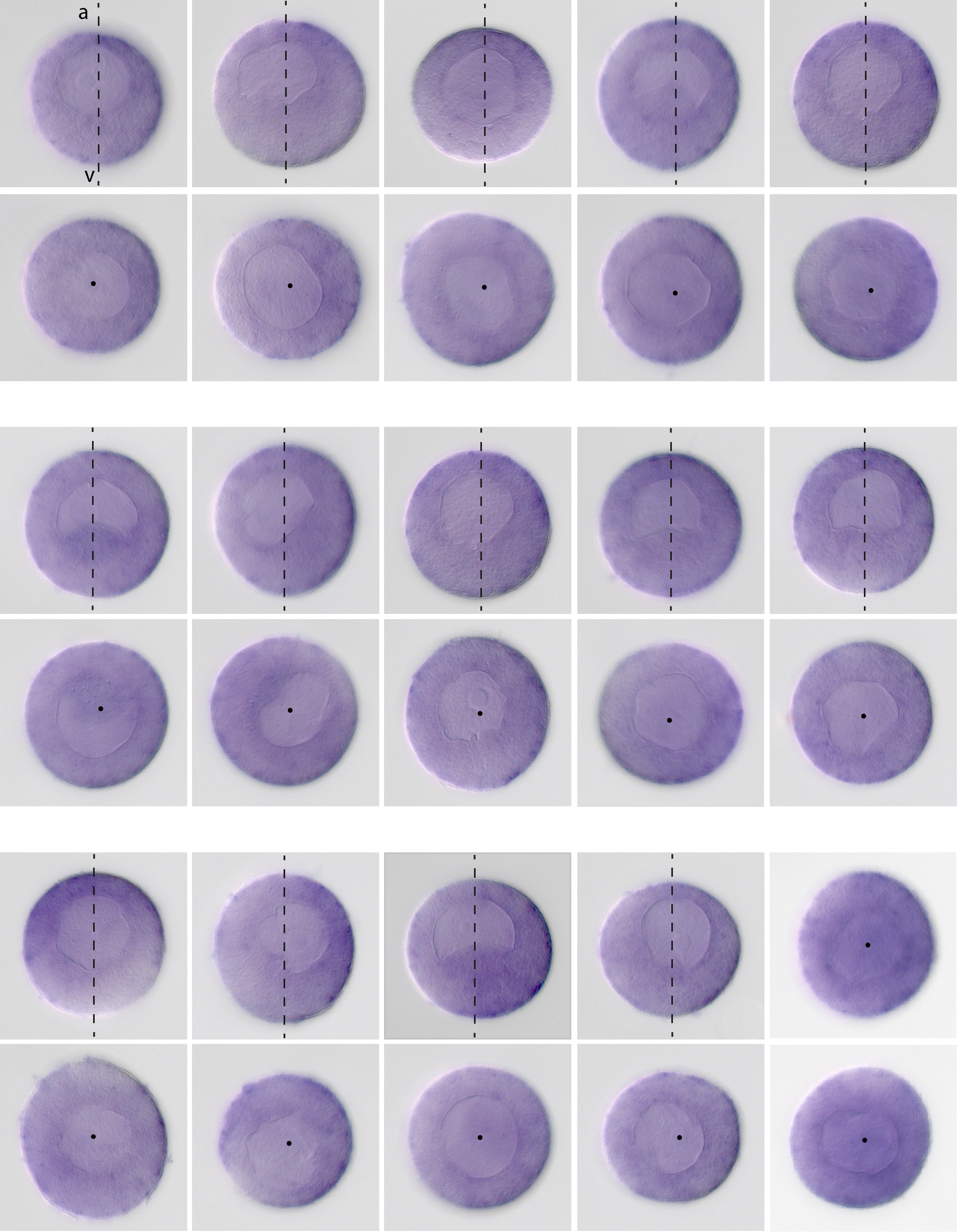

Supplement: S9 Fig — Immature oocytes were collected from gonads and analyzed for the expression of panda by in situ hybridization. Results are presented for a randomly selected set of 16 ovocytes. The position of the animal-vegetal axis of the ovocyte is revealed by the asymmetrical localization of the germinal vesicle. The germinal vesicle and its nucleolus are closer to the animal pole where the micropyle and polar bodies will form [39]. When possible, for each ovocyte, a lateral view showing the animal-vegetal axis as a dashed line (top image) and an axial view showing it as a dot (lower image) are shown. Note, however, that at this stage it is not possible to recognize the future D/V embryonic axis. The subcortical localization of the in situ hybridization signal suggests that panda mRNA could be localized to the egg cortex. (TIF) [file pbio.1002247.s010.tif]
